# Brain metastases exhibit distinct spatial patterns of resident and infiltrating macrophages

**DOI:** 10.1038/s41420-026-03084-0

**Published:** 2026-04-01

**Authors:** Avinoam Ratzabi, Itai M. Caspit, Ira Telechi, Jung-Seok Kim, Hananya Vaknine, Pablo Blinder, Steffen Jung, Reuven Stein

**Affiliations:** 1 Department of Neurobiology, School of Neurobiology, Biochemistry and Biophysics, George S. Wise Faculty of Life Sciences, Tel Aviv University, Tel Aviv, Israel; 2 Sagol School of Neuroscience, Tel Aviv University, Tel Aviv, Israel; 3 Department of Immunology and Regenerative Biology, Weizmann Institute of Science, Rehovot, Israel; 4 Institute of Pathology, E. Wolfson Medical Center, Holon, Israel

**Keywords:** CNS cancer, Neuroimmunology

## Abstract

Brain metastases (BrM) are a leading cause of morbidity and mortality, arising in multiple brain compartments. BrM colonization and progression are shaped by interactions with distinct tumor-associated macrophage (TAM) subsets, including microglia (MG), monocyte-derived macrophages (MDM), and border-associated macrophages (BAM). While transcriptomes of TAM have been characterized in detail, their spatial distribution, abundance, and compartment-specific composition -particularly in relation to BrM size and the cancer origin—remain poorly defined. Here, we performed a comprehensive spatial analysis of TAM subtypes across brain regions using experimental BrM models of lung and breast cancer, as well as melanoma. We distinguished TAM subsets by both origin and location, employing genetically traceable mouse models. We observed expansion of MG and BAM, as well as MDM infiltration associated with BrM, albeit with distinct compositions. Parenchymal BrM contained both MG and MDM, whereas ventricular and leptomeningeal BrM contained BAM and MDM but lacked MG. TAM abundance varied with BrM size, compartment, and cancer type: MG predominated in early parenchymal lesions, with MDM becoming dominant as tumors grew. Notably, melanoma BrM exhibited markedly reduced MDM infiltration compared with lung and breast cancer BrM. These findings highlight the need to tailor TAM-targeted therapies not only to the primary tumor type but also to the brain compartment affected. A deeper understanding of TAM dynamics across compartments may improve the precision and efficacy of BrM treatments.

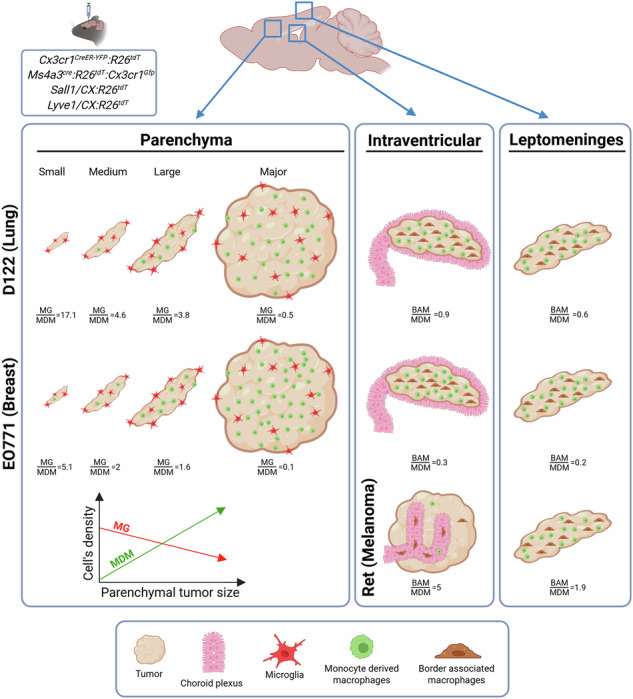

## Introduction

Up to a quarter of cancer patients develop brain metastases (BrM), with lung, breast, and melanoma cancers having the highest incidence [1–3]. BrM are associated with high morbidity and unfavorable prognosis with a median survival of less than one year [4, 5]. They often form within the brain parenchyma but can also develop in the meninges, i.e., dura and/or leptomeninges, or inside the brain ventricles [6–8].

The growth of primary and metastatic tumors depends on interactions between cancer cells and their tumor microenvironment (TME), comprising stromal and immune cells [9, 10]. Notably, the CNS milieu differs substantially from that of peripheral tissues where primary tumors arise, as it comprises neurons and glial cells, including astrocytes and microglia (MG)—the resident parenchymal macrophages of the CNS. MGs detect and respond to damaging signals, such as tumors, through rapid activation, adopting a broad spectrum of states that range from pro- to anti-inflammatory, including disease-associated phenotypes [11–15] that lead to neuroinflammation [16]. Neuroinflammation is not restricted to the action of glia, but under pathological conditions, circulating blood monocytes infiltrate the brain and differentiate into monocyte-derived macrophages (MDM) [17, 18]. Tumor-associated (TA) MG and MDM, often referred to as tumor-associated macrophages (TAM), are the most abundant nontumoral cell types found in the TME associated with BrM [19–21]. The composition of the brain microenvironment, including TAM and other parenchymal cells such as astrocytes, has been shown to influence the development of BrM [16, 18, 22–28].

Besides the parenchymal MG, resident CNS macrophages also include a group of macrophages that reside in the CNS borders [meninges, perivascular spaces, and choroid plexus (CP)], collectively termed border-associated macrophages (BAM) [29–32]. BAM represents a distinct macrophage population whose features and potential functions are shaped by both their location and ontogeny. Meningeal BAM comprises dural and leptomeningeal cells that have been associated with BrM [33–35], although little is known about their contributions in BrM.

Flow cytometry and single-cell RNA sequencing have yielded valuable insights into the roles of the TME in human and mouse BrM, including signaling pathways that regulate tumor progression, as well as patient fates and responsiveness to immunotherapy [36]. Although these studies have provided critical insights, they have limitations when it comes to the study of TAM. First, the expression of macrophage markers, such as CX3CR1, CD45, P2RY12, and TMEM119, is dynamic under pathological conditions, including brain tumors [33, 37, 38]. Consequently, bioinformatic analyses may have missed diverse cell origins and failed to distinguish whether different TAM subsets represent distinct cell types or states. Moreover, previous studies largely lacked spatial information regarding TAM composition, localization, physical interactions, and their relationship to tumor growth. In fact, histological analyses suffer from the absence of reliable markers that allow robust discrimination of macrophage subtypes, such as MG, BAM, and MDM.

In this study, we focused on characterizing the spatial distribution, composition, and interactions of distinct macrophage subtypes with BrM and other cells of the TME in experimental metastases across different brain compartments. To discriminate among TAM subtypes, we employed four complementary lineage-tracing mouse models. Since the macrophage landscape of BrM is shaped by the type of cancer cells from which the metastases originate [19, 20, 23, 39], we examined multiple prevalent BrM cancer types: lung cancer, breast cancer, and melanoma, represented by D122 Lewis lung carcinoma [40], EO771 breast cancer [41], and Ret melanoma [42], respectively.

We show that the TAM landscape in BrM is significantly shaped by both the CNS compartment and the type of primary cancer. A common feature across all brain compartments was that TAM comprised a mixture of resident macrophages and infiltrating MDM. However, TAM composition differed by location: parenchymal BrM were primarily populated by MG and MDM, whereas leptomeningeal and lesions localized to the CP (CP BrM; CP-BrM) contained BAM and MDM. Our results further indicate that MG are the predominant myeloid cell type in small, perivascular-like parenchymal BrM, whereas MDM dominate in larger BrM. Importantly, both MG and MDM are heterogeneous in BrM-bearing brains.

## Results

### Metastatic distribution patterns across the BrM experimental mouse models used

**Fig. 1:**
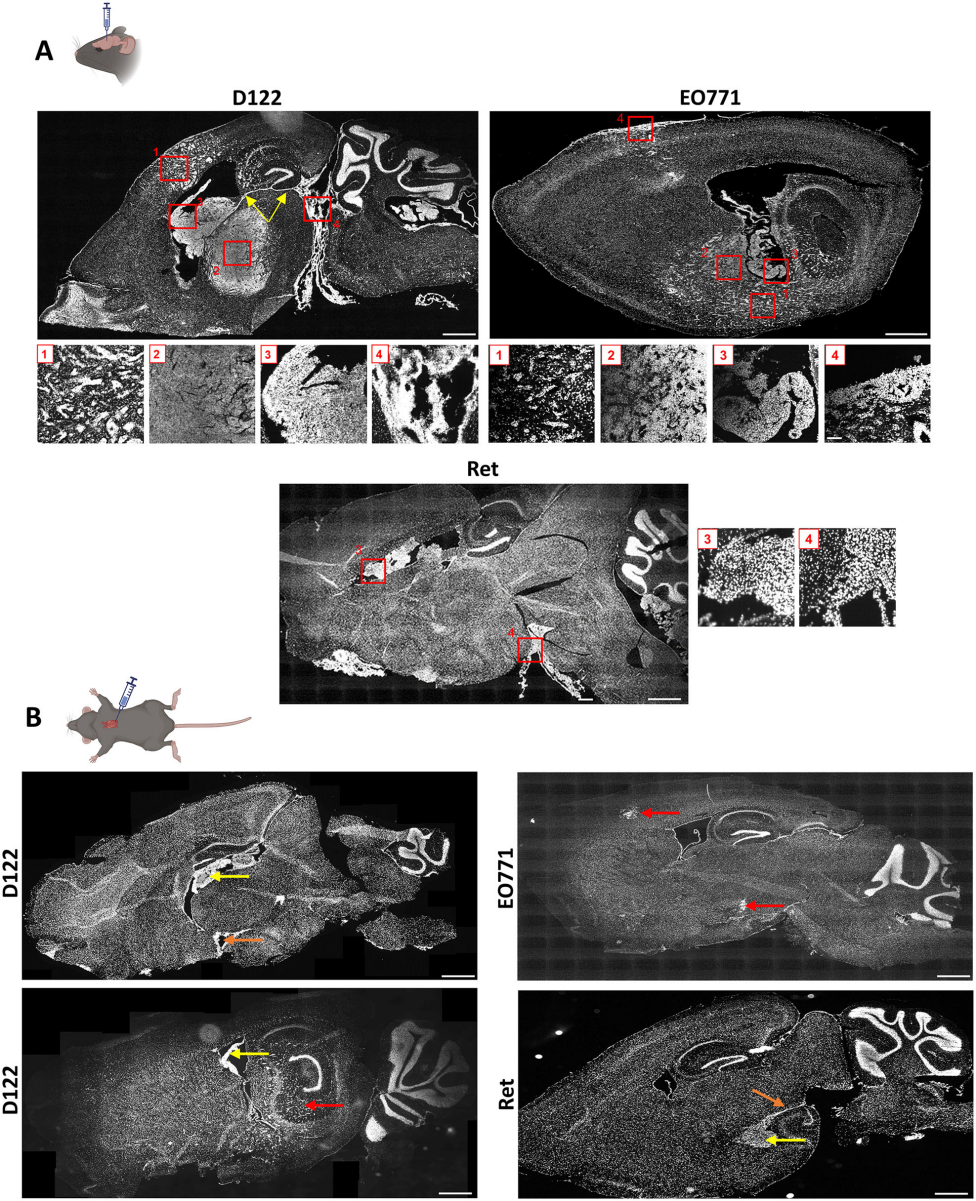
Colonization patterns of experimental BrM from lung cancer, breast cancer, and melanoma were established in mice by intracranial or intracardiac injection. D122, EO771, or Ret cells were injected into *Cx3cr1*^*CreER-YFP*^*:R26*^*tdT*^ mice. Shown are representative scanned sagittal sections stained with DAPI to visualize metastatic lesions for each cancer type. **A** Intracranial injection. Red boxes highlight four distinct BrM growth patterns: parenchymal [microtumors (1) and major tumors (2)], ventricular (3), and leptomeningeal (4). Scale bars, 1000 µm. Enlarged views of the boxed regions are displayed below the corresponding sagittal sections (scale bars, 100 µm). Yellow arrows in the D122 BrM-containing section mark the internal meningeal substructure that extends beneath the hippocampus and penetrates the ventricular CP. **B** Intracardiac injection. Red arrows indicate parenchymal microtumors, yellow arrows ventricular BrM, and orange arrows leptomeningeal BrM. Scale bar: 1000 µm.

BrM were established utilizing experimental models of the three prevalent cancer types by intracranial or intracardiac implanting D122 Lewis lung carcinoma, EO771 breast cancer, or Ret melanoma cells (Fig. S1A). BrM lesions were identified by high cell nuclei content, indicated by DAPI staining (Fig.1). The DAPI signal in these regions colocalized with a Ki-67 signal indicative of cell proliferation (Fig. S2A). Moreover, tumors identified by hematoxylin and eosin (H&E) staining displayed distribution patterns and architecture comparable to those observed with DAPI staining (Fig. S2B), confirming that DAPI staining is a reliable method for BrM detection.

Intracranial injections of all cancer types led to the generation of BrM in the parenchyma, ventricle, and leptomeninges (Fig.1A, inserts 1–4). The tumor architecture, however, varied by cancer type. In D122 and EO771 models, parenchymal BrM lesions developed in ~95% of injected mice and exhibited two main distribution patterns: clusters of small foci of varying sizes (hereafter referred to as microtumors) (insert 1), and a larger tumor mass (insert 2), likely formed by the coalescence of multiple microtumors, though still partially separated at the tumor boundary. In the Ret melanoma model, only ~17% of mice developed parenchymal tumors, which were largely restricted to the injection site. Intraventricular or leptomeningeal tumors (insert 3 and 4) were detected in ~70–100% and ~50–80% of injected mice, respectively. Leptomeningeal BrM from all cancer types arose in diverse locations, including the dorsal and/or ventral brain surface (external leptomeninges), as well as internal meningeal structures, such as the substructure extending beneath the hippocampus and penetrating the ventricular CP (e.g., Fig. 1A, yellow arrows).

Intracardiac injection of D122 cells yielded metastatic lesions mainly in the ventricle (Fig. 1B); however, some small parenchymal microtumors and leptomeningeal lesions were also observed. On the other hand, the majority of EO771 metastatic lesions appeared as small parenchymal microtumors and rarely also in the leptomeninges. Ret metastatic lesions were mainly found in the ventricles and in a few cases also in the leptomeninges. Notably, only a low percentage of the intracardiac-injected mice developed BrM, and these metastases were very small. This likely reflects the dissemination of tumor cells to multiple extracranial sites, which caused mortality before substantial BrM could develop. Consequently, in subsequent experiments, we focused on BrM generated by intracranial injection.

### Spatial distributions of tumor-associated MG and MDM in parenchymal BrM

The parenchymal BrM formed by D122 and EO771 cells, encompassing microtumors and major tumors of varying sizes, likely represents distinct stages of parenchymal BrM progression. To gain insight into the role of TAM in this process, we examined the relationship between tumor size and the spatial distribution and content of TA-MG and MDM. To this end, we categorized parenchymal BrM lesions into four subgroups according to their size: a major tumor (>338,511 µm^2^) and three microtumor subgroups (small: 130–418 µm^2^, medium: 643–1287 µm^2^, and large: 1931–3219 µm^2^) (Fig. S1B). Then, we compared the spatial distribution of MG and MDM in the different BrM regions and the relative MG and MDM distribution in distal nontumor regions. BrM were established in *Cx*_*3*_*cr1*^*CreER-YFP*^*:R26*^*tdT*^ mice, which enable discrimination of MG and MDM [43]. In these animals, MG are labelled by tdT and YFP, whereas MDM are labelled only by YFP. Notably, in some cases, the YFP signal [identified by anti-GFP immunofluorescence (IF) staining] was low, probably because of low *Cx3cr1* expression. Thus, to ensure that all MG and MDM were detected, we used Iba1 as an additional MG and MDM marker.

**Fig. 2:**
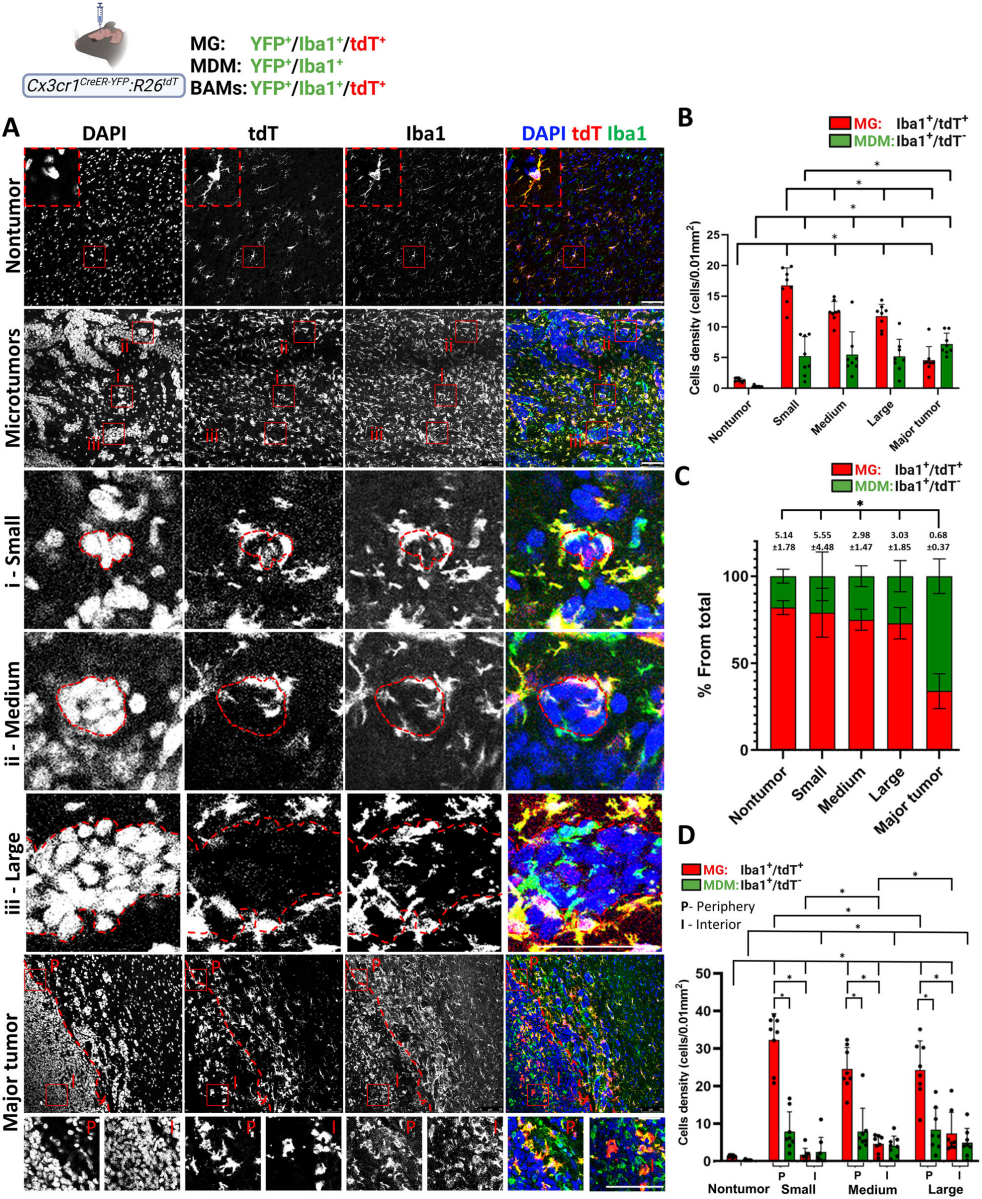
Spatial distribution of tumor-associated MG and MDM in D122 parenchymal BrM. *Cx3cr1*^*CreER-YFP*^*:R26*^*tdT*^ mice were intracranially injected with D122 cells. Sagittal sections were stained with DAPI and anti-Iba1. **A** Representative confocal IF image showing TA-MG and MDM distribution in nontumor, microtumor, and major tumor regions (*n* = 8 mice/subgroup). Individual channels (DAPI, tdT, Iba1) and merged images are shown. The inset in the nontumor panel shows an enlarged view of the red boxed region, highlighting a representative MG. In the microtumor panel, red boxes indicate (i) small, (ii) medium, and (iii) large microtumors, with corresponding enlargements shown in the panels below. Dotted lines delineate tumor borders. TA-MG accumulates both around and at the surface of the microtumors. In the major tumor, boxes indicate interior (I) and periphery (P), with TA-MG present in both regions. Scale bar, 75 μm. **B** Quantification of MG (tdT⁺/Iba1⁺) and MDM (tdT^−^/Iba1⁺) densities in nontumor and tumoral regions of parenchymal BrM subgroups. Densities were measured across the entire tumoral region, as described in the “Materials and methods” section. Data are presented as cell density (cells/0.01 mm²), mean ± SEM (*n* = 8 mice). Dots represent individual mice. ^*^q < 0.05. **C** Relative fractions of MG and MDM from total Iba1^+^ cells within each of the indicated regions, presented as subdivided bar graphs (mean ± SEM; *n* = 8). MG/MDM ratios are indicated above the bars, and statistical analysis was performed on these ratio values. ^*^q < 0.05. **D** Quantification of TA-MG and MDM densities in the periphery (P) and interior (I) of the microtumor subgroups. Densities were determined as described in the “Materials and methods” section and are presented as cell density (cells/0.01 mm²), mean ± SEM (*n* = 8 mice). For reference, the densities of MG and MDM in the nontumoral region (**B**) are included. Dots represent individual mice. ^*^q < 0.05.

Next, we assessed the spatial distribution of TA-MG (tdT⁺/Iba1⁺) and MDM (tdT^−^/Iba1⁺; Iba1-only) in parenchymal D122 microtumors and major tumors (Fig. 2A). Quantification of TA-MG and MDM densities across the different tumor subgroups revealed significantly higher densities of both cell types in all tumor subgroups compared with nontumor tissue (Fig. 2B). TA-MG density was highest in small microtumors and gradually declined as tumor size increased (Spearman correlation in R, *p* = 0.041). In contrast, MDM density was significantly greater in major tumors than in microtumors. Thus, with tumor progression, the proportional composition of TAM shifted from TA-MG predominance toward an increasing contribution of MDM (Fig. 2C).

To further dissect the spatial distribution of TA-MG and MDM within microtumors, we quantified their densities in the tumor periphery and core across the different subgroups. In all microtumor subgroups, TA-MG were abundant at the tumor periphery, with significantly higher densities compared to both nontumor regions and the corresponding tumor cores (Fig. 2D). As tumors grew, TA-MG density at the periphery decreased, while their density in the core increased. Some MDM were detected at the tumor periphery, although their density was much lower than that of TA-MG (Fig. 2D). Within the cores of small, medium, and large microtumors, MDM density was low but significantly higher than in nontumor regions, indicating a modest level of MDM infiltration into microtumors.

Collectively, these findings suggest that in the D122 model, TA-MG constitute the primary macrophage subtype initially responding to the emergence of parenchymal tumors. TA-MG are first recruited to the tumor periphery, and with tumor growth, some TA-MG infiltrate into the core. In parallel, MDM gradually accumulates within the tumor cores, ultimately becoming the predominant macrophage population in major tumors.

**Fig. 3:**
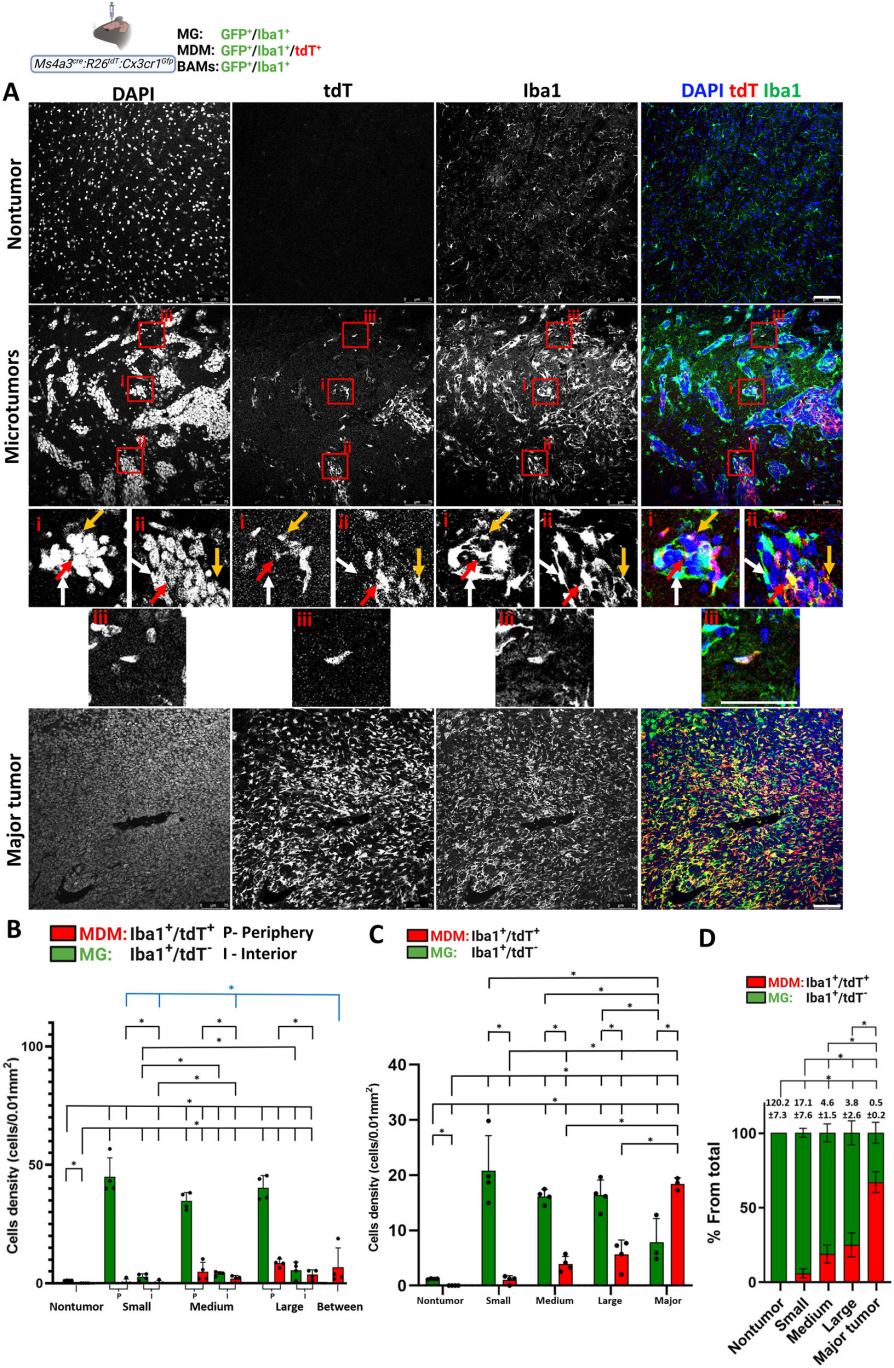
Spatial distribution of tumor-associated MG and MDM in parenchymal D122 BrM using *Ms4a3*^*cre*^*:R26*^*tdT*^*:Cx3cr1*^*Gfp*^ mice. Mice were intracranially injected with D122 cells; sagittal sections were stained with DAPI and anti-Iba1. **A** Representative confocal IF image showing TA-MG (tdT^−^/Iba1⁺) and MDM (tdT⁺/Iba1⁺) in nontumor, microtumor, and major tumor regions (*n* = 4 mice). Insets show small (i), large (ii), and intertumoral (iii) regions. Arrows indicate MDM at the tumor interior (red) and MDM (yellow) or TA-MG (white) at the tumor surface. Scale bar, 75 μm. **B** Quantification of TA-MG and MDM densities in nontumor, microtumor periphery (P) and interior (I), and intertumoral (between) regions (cells/0.01 mm²; mean ± SEM; *n* = 4). Dots, individual mice. ^*^q < 0.05. Comparisons to nontumor shown in black; between-microtumor vs. subgroups in blue. **C** Quantification of TA-MG and MDM densities in nontumor tissue (same samples as in **B**) and across the entire tumor regions of the different BrM subgroups (cells/0.01 mm²; mean ± SEM; *n* = 4 for microtumors, *n* = 3 for major tumors). **D** Relative fractions of MG and MDM among total Iba1⁺ cells. Bars show mean ± SEM (*n* = 4 for microtumors, *n* = 3 for major). MG/MDM ratios are indicated above the subdivided bars; statistics are performed on ratio values. ^*^q < 0.05.

To validate the findings obtained with *Cx*_*3*_*cr1*^*CreER-YFP*^*:R26*^*tdT*^ animals, we used a complementary lineage-tracing model, *Ms4a3*^*cre*^*:R26*^*tdT*^*:Cx3cr1*^*Gfp*^ mice [44, 45], in which all brain macrophages express *Cx3cr1*-GFP, whereas monocyte-derived cells, but not yolk sac–derived MG, are permanently labeled with tdT [46], thereby allowing direct identification of MDM by their tdT expression. The results obtained with the intracranially injected D122 model were consistent with those observed in *Cx*_*3*_*cr1*^*CreER-YFP*^*:R26*^*tdT*^ mice. Microtumor-associated MG (GFP⁺/Iba1⁺, tdT^−^) were predominantly localized around the tumors, with some cells present within the core, where their density modestly increased as tumors expanded (Fig. 3A, B). TA-MG also populated the major tumors (Fig. 3A, C). MDM (tdT⁺) density in microtumors was low, but increased with tumor growth (Fig. 3C), and within microtumors, abundance was higher at the periphery over the core (Fig. 3A, B). Notably, MDM also appeared in the spaces between microtumors (Fig. 3B). Finally, as in *Cx*_*3*_*cr1*^*CreER-YFP*^*:R26*^*tdT*^ mice, the MG/MDM composition shifted from TA-MG predominance in microtumors toward MDM dominance in major tumors (Fig. 3C, D).

**Fig. 4:**
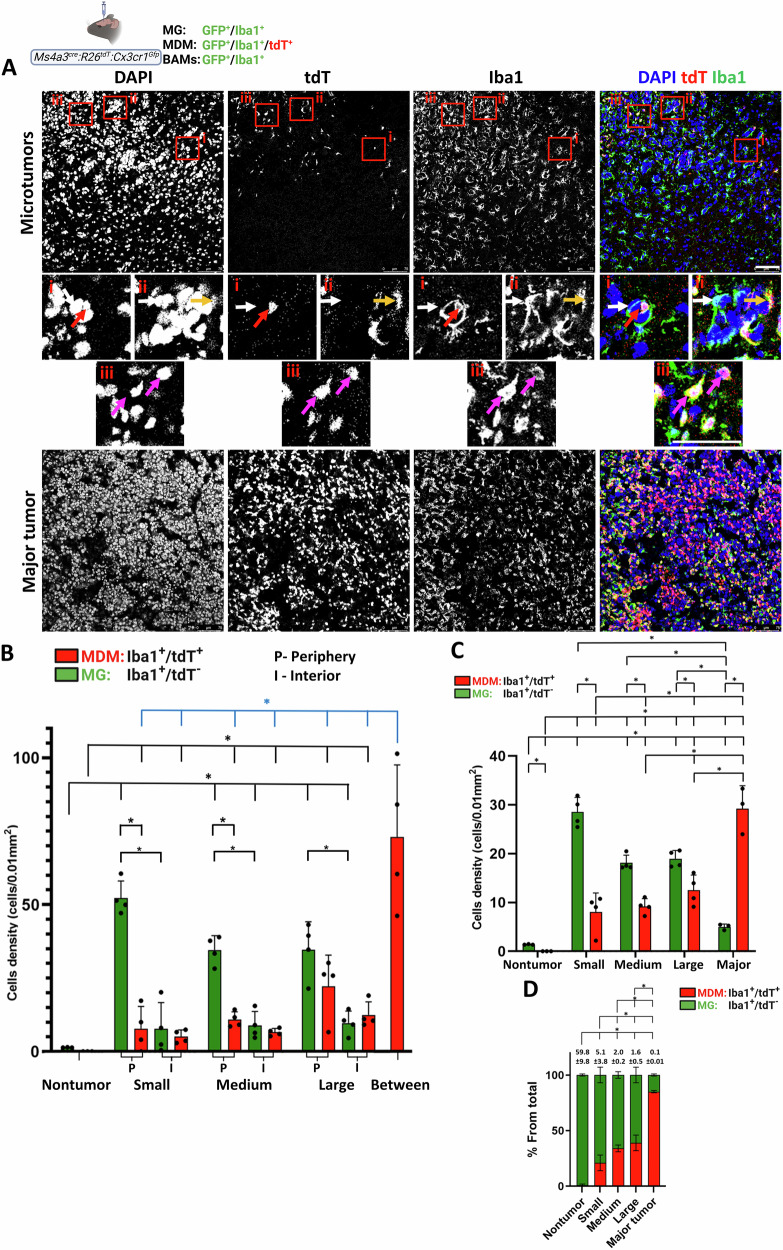
Spatial distribution of tumor-associated MG and MDM in parenchymal EO771 BrM using *Ms4a3*^*cre*^*:R26*^*tdT*^*:Cx3cr1*^*Gfp*^ mice. Mice were intracranially injected with EO771 cells; sagittal sections were stained with DAPI and anti-Iba1. Data were analyzed and presented as in Fig. 3. **A** Representative confocal IF images showing the distribution of MG and MDM across nontumor, microtumor, and major tumor regions. Pink arrows in enlargement (iii) highlight MDM in the intertumoral region. (*n* = 4 mice) **B** Quantification of TA-MG and MDM densities in nontumor, microtumor P/I, and intertumoral (between) regions. **C** Quantification of densities in nontumor and across entire tumor regions. **D** Relative fractions of MG and MDM among total Iba1⁺ cells. MG/MDM ratios indicated above bars. *n* = 4 for microtumors, *n* = 3 for major tumors. ^*^q < 0.05.

Next, we analyzed the distribution of TA-MG and MDM in parenchymal EO771 BrM in *Ms4a3*^*cre*^*:R26*^*tdT*^*:Cx3cr1*^*Gfp*^ mice. Overall, the results in the EO771 model resembled those obtained with the D122 model in terms of the spatial distribution and composition of TA-MG and MDM in both microtumors and the major tumor (Fig. 4A–D). Specifically, we observed extensive accumulation of TA-MG at the microtumor periphery (Fig. 4A, B), a predominance of TA-MG over MDM within microtumors (Fig. 4B–D), and a gradual shift toward MDM dominance in the major tumor (Fig. 4D). In addition, in both BrM models, MDM were also localized at the microtumor periphery and in the spaces between microtumors (Fig. 4A, B). However, the models differed in their MDM responses: in the EO771 model, both the fraction of MDM within the total TAM population (Fig. 4D) and their density between microtumors were higher than in the D122 model (Figs. 4B; cf. 3B).

Taken together, the results from both lineage-tracing mouse models indicate that in D122 and EO771 BrM, MG represent the predominant TAM population responding to parenchymal BrM at early stages, whereas the relative contribution of MDM increases as tumors expand. The results also show that, in addition to infiltrating the tumor core, MDM, particularly in EO771 BrM, are recruited to the microtumor periphery and to intertumoral regions.

### The effect of BrM on TA-MG and MDM

**Fig. 5:**
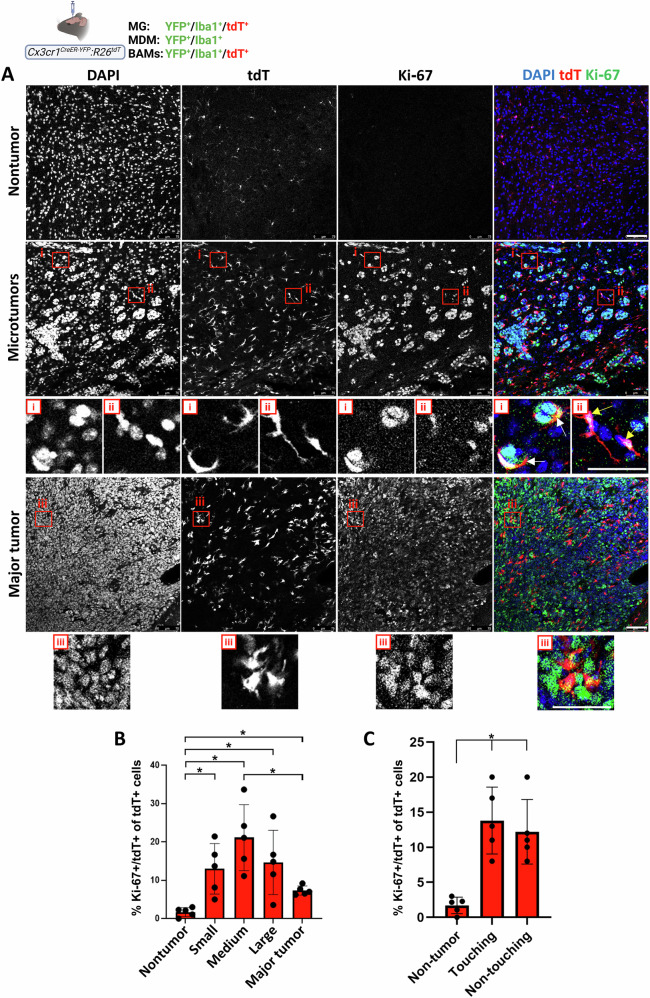
MG proliferation in nontumor and parenchymal BrM regions. *Cx3cr1*^*CreER-YFP*^*:R26*^*tdT*^ mice were intracranially injected with D122 cells; sagittal sections were stained with DAPI and anti–Ki-67. **A** Representative confocal IF image showing DAPI, tdT, and Ki-67 signals. Individual channels and corresponding merged images are presented (*n* = 5). Boxed regions highlight proliferating MG (tdT^+^/Ki-67^+^) in small microtumors (i), between microtumors (ii), and inside major tumors (iii); enlargements are shown below. Scale bar, 75 μm. **B**, **C** Quantification of proliferating MG as a percentage of total MG (mean ± SEM; *n* = 5). **B** Frequencies in nontumor and BrM subgroups. **C** Frequencies of proliferating MG in direct (white arrows) or indirect (yellow arrows) contact with microtumors, compared with nontumor MG. ^*^q < 0.05.

Given the accumulation of TA-MG in the parenchymal BrM region, we next asked whether this was accompanied by increased MG proliferation using *Cx3cr1*^*CreER-YFP*^*:R26*^*tdT*^ mice intracranially implanted with D122 tumor cells. MG proliferation was assessed by Ki-67 staining (Fig. 5A). The percentage of proliferating MG was significantly higher in all parenchymal BrM subgroups compared to MG in nontumor regions (Fig. 5B). Among BrM subgroups, proliferation rates were largely similar, except for a modest increase in medium-sized microtumors relative to major tumors (Fig. 5B). Proliferating TA-MG were frequently observed in direct contact with microtumors, including very small lesions of only 2–4 tumor cells (Fig. 5Ai), but were also detected in intertumoral areas lacking direct tumor contact (Fig. 5Aii, C). These findings suggest that local MG proliferation contributes to MG accumulation around and within BrM lesions, and that direct MG-cancer cell interactions are not essential to induce MG proliferation. Notably, only approximately 1% of the MDM population was Ki-67 positive (Fig. S3), indicating that monocyte infiltration, rather than local proliferation, is the main source of MDM within BrM.

**Fig. 6:**
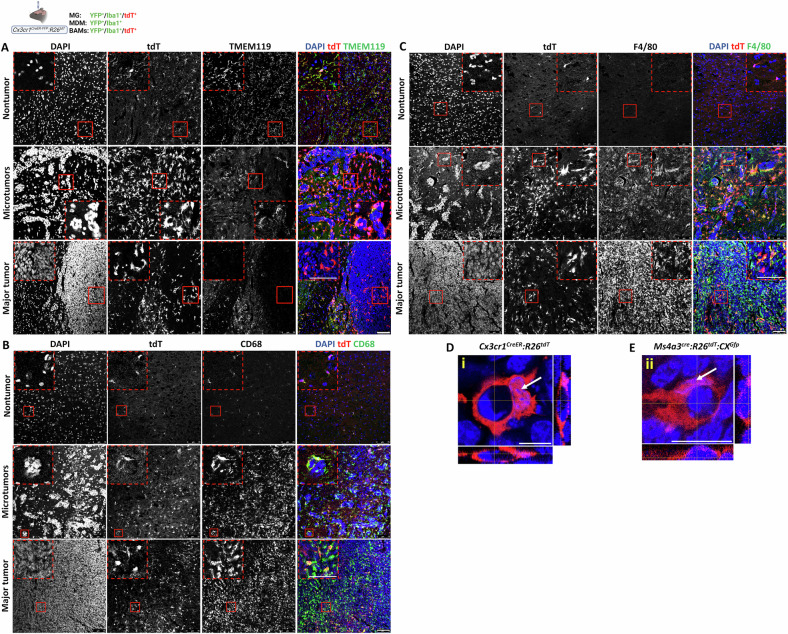
Marker expression and phagocytic activity in tumor-associated MG and MDM. *Cx3cr1*^*CreER-YFP*^*:R26*^*tdT*^ mice were intracranially injected with D122 cells. **A**–**C** Confocal IF images showing tdT and TMEM119 (**A**), CD68 (**B**), or F4/80 (**C**) signals in the indicated regions (n = 6–8). Insets show boxed enlargements. Scale bar, 75 μm. **D**, **E** Phagocytic activity in D122 BrM. Confocal z-stacks from *Cx3cr1*^*CreER--YFP*^*:R26*^*tdT*^ (**D**) or *Ms4a3*^*cre*^*:R26*^*tdT*^*:Cx3cr1*^*Gfp*^ showing representative TA-MG (**D**) and MDM (**E**) engulfing pyknotic nuclei. Maximum intensity projections with orthogonal views at the crosshair level demonstrate nuclear internalization. White arrows indicate the MG or MDM nucleus. Scale bar, 15 μm.

The effect of BrM on TA-MG properties was also examined by assessing the expression of the MG signature protein TMEM119 [47] and the macrophage activation markers F4/80 and CD68. TMEM119 was expressed by MG in nontumor and intertumoral regions but was markedly downregulated in TA-MG (Fig. 6A). This reduction was most pronounced in TA-MG within major tumors, where only a few cells retained detectable TMEM119, and was also observed, albeit to a lesser degree, in MG associated with microtumors. In contrast, expression of CD68 (Fig. 6B) and F4/80 (Fig. 6C) was elevated in most TA-MG compared with MG in nontumor regions, both in microtumors-associated TA-MG and in those within major tumors. As expected, F4/80 and CD68 were expressed in MDM (F4/80^+^tdT^−^ and CD68^+^tdT^−^ cells) across the different tumor subtypes (Fig. 6B, C). Notably, at least a subset of TA-MG and TA-MDM exhibited phagocytic activity, as indicated by evidence of cell engulfment (Fig. 6D). Taken together, these results suggest that the presence of BrM promotes MG proliferation and shifts their protein expression profile toward a macrophage-like phenotype.

### Identification of MG and MDM subsets in BrM by flow cytometry

**Fig. 7:**
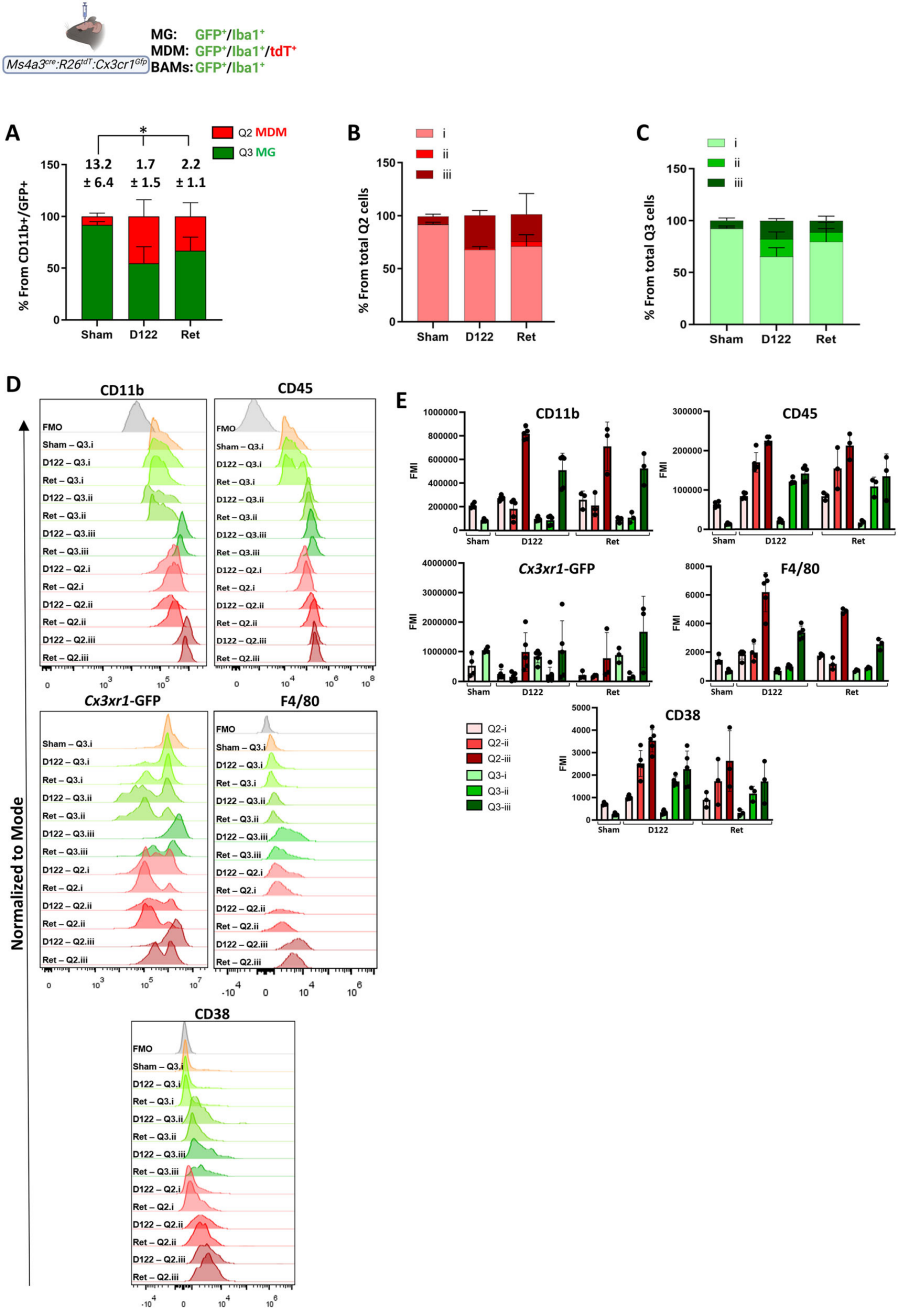
Flow cytometric characterization of macrophage subpopulations in brains bearing metastases. Brains from *Ms4a3*^*cre*^*:R26*^*tdT*^*:Cx3cr1*^*Gfp*^ mice injected with D122 cells, Ret cells, or sham controls were processed for flow cytometry at 21, 14, and 21 dpi, respectively (see “Materials and Methods” section). CD11b⁺ cells were gated based on tdT and GFP expression. The resulting MDM (tdT⁺/GFP⁺) and MG (tdT⁻/GFP⁺) populations—corresponding to Q2 and Q3 gates in Fig. S4B—were further subdivided by CD11b and CD45 expression into three subpopulations: (i) CD45^int^/CD11b^int^, (ii) CD45^hi^/CD11b^int^, and (iii) CD45^hi^/CD11b^hi^. **A** Quantification of MDM (Q2) and MG (Q3) as a percentage of total CD11b⁺/Cx3cr1-GFP⁺ cells in sham, D122-, and Ret-BrM brains. Data represent mean ± SEM (*n* = 5, 5, and 3 for sham, D122, and Ret, respectively). The MG/MDM ratio was calculated per mouse, and mean ± SEM values are indicated above the subdivided bars. Statistical analysis was performed on ratio values (*q* < 0.05). **B**, **C** Quantification of subpopulations (i–iii) within MDM (Q2, **B**) and MG (Q3, **C**) populations, shown as subdivided bar graphs (mean ± SEM). **D**, **E** Expression of CD11b, CD45, Cx3cr1-GFP, F4/80, and CD38 in MDM (Q2) and MG (Q3) subpopulations from sham, D122-, and Ret-BrM brains. **D** Representative normalized histograms (normalized to model); FMO fluorescence minus one. **E** Quantification of protein expression as fluorescence median intensity (FMI; background-subtracted FMO). Data represent mean ± SEM; dots indicate individual mice (*n* = 5, 5, and 3 for sham, D122, and Ret, respectively).

To further assess TAM properties in BrM, we employed whole-brain flow cytometry in D122- and Ret-BrM–bearing *Ms4a3*^*cre*^*:R26*^*tdT*^*:Cx3cr1*^*Gfp*^ mice, with sham and blood samples as controls. CD11b⁺ cells were separated by reporter expression into MDM [GFP⁺/tdT⁺, quadrant 2 (Q2)] and MG [GFP⁺/tdT⁻, quadrant 3 (Q3)] (Fig. S4A, B). Each population was further subdivided into three subgroups (i–iii) according to CD11b/CD45 expression levels (Fig. S4C). BrM-bearing brains contained a higher proportion of MDM than sham brains (Fig. 7A) and displayed distinct shifts in the relative abundance of both MDM and MG subgroups compared with shams (Fig. 7B, C, respectively). Within the MDM population, the relative abundance of the Q2/i subgroup was reduced, whereas the Q2/ii and especially the Q2/iii-subgroups were increased in both D122- and Ret-BrM brains compared with sham controls. Similarly, in the MG population, the relative abundance of the Q3/i subgroup was reduced, while Q3/ii and Q3/iii subgroups were increased, more prominently in D122 BrM and to a lesser extent in Ret BrM brains (Fig. 7B, C and Table 1).

**Table 1:** Percentage of MDM (Q2) and MG (Q3) subpopulations (Q2 i–iii and Q3 i–iii) in sham-, D122-, and Ret-injected brains of *Ms4a3*^*cre*^*:R26*^*tdT*^*:Cx3cr1*^*Gfp*^ mice, as quantified by flow cytometry.

	Q2 (MDM)			Q3 (MG)		
	Q2i	Q2ii	Q2iii	Q3i	Q3ii	Q3iii
Sham	91.60%	0.12%	7.46%	92.40%	0.44%	7.23%
D122	67.86%	1.39%	31.06%	65.30%	16.71%	17.76%
Ret	70.28%	5.25%	24.75%	75.60%	12.58%	11.14%

To further characterize the properties of the MG and MDM subpopulations, we assessed the expression of CD11b, CD45, *Cx3cr*1-GFP, F4/80, and CD38 in each subgroup (Fig. 7D, E). The analysis revealed distinct marker expression patterns in the different MDM and MG subgroups that probably reflect different differentiation or activation states of MDM and MG, respectively (Table 2). Accordantly, Q2/i likely corresponds to monocytes or monocyte-like cells, while Q2/ii and Q2/iii represent intermediate and differentiated macrophages, respectively. Similarly, Q3/i corresponds to non-activated MG, whereas Q3/ii and Q3/iii represent activated MG at different activation states.

**Table 2:** Expression levels of CD11b, CD45, *Cx3cr1*-GFP, F4/80, and CD38 in MDM (Q2) and MG (Q3) subpopulations (i–iii) from sham- D122-, and Ret-BrM brains, as assessed by flow cytometry.

		CD11b	CD45	Cx3cr1-GFP	F4/80	CD38
MDMQ2i	Sham	**Int** (209,684)	**Int** (63,344)	**Int** (522,040)	**Int** (1442)	**Int**/− (708)
	D122	**Int** (274,836)	**Int** (84,354)	**Int** (241,178)	**Int** (1829)	**Int**/− (1006)
	Ret	**Int** (257,368)	**Int** (82,977)	**Int** (192,643)	**Int** (1755)	**Int**/− (895)
MDM Q2ii	D122	**Int** (184,832)	**High** (170,208)	**Low** (159,418)	**Int** (1960)	**Int** (2514)
	Ret	**Int** (190,568)	**High** (142,392)	**Low** (182,774)	**Int** (1170)	**Int** (1717)
MDM Q2iii	D122	**High** (819,400)	**High** (225,284)	**High** (986,876)	**High** (6192)	**High** (3526)
	Ret	**High** (686,500)	**High** (202,648)	**High** (727,166)	**High** (4865)	**High** (2625)
MGQ3i	Sham	**Low** (84,982)	**Low** (13,584)	**High** (1,046,750)	**Low** (647)	**Low** (241)
	D122	**Low** (98,554)	**Low** (21,413)	**High** (833,200)	**Low** (689)	**Low** (337)
	Ret	**Low** (88,041)	**Low** (16,205)	**High** (854,000)	**Low** (701)	**Low** (313)
MGQ3ii	D122	**Low** (87,032)	**High** (121,399)	**Low** (220,206)	**Low** (984)	**High** (1714)
	Ret	**Low** (96,857)	**High** (102,507)	**Low** (168,644)	**Low** (881)	**High** (1175)
MGQ3iii	D122	**High** (511,333)	**High** (141,892)	**High** (1,042,506)	**High** (3361)	**High** (2265)
	Ret	**High** (482,428)	**High** (131,354)	**High** (3,312,806)	**High** (2553)	**High** (1713)

The table presents the mean fluorescence intensity (MFI) values corresponding to Fig. 7E. The relative annotations low, int, and high refer to the expression level of each protein across the different macrophage subtypes in sham-, D122-, and Ret-BrM-bearing brains. The analysis reveals the following classification:

MDM: Q2/i, (CD11b^int^/CD45^int^/*Cx3cr1*-GFP^int^/F4/80^int^/CD38^int/−^); Q2/ii, (CD11b^int^/CD45^high^/*Cx3cr1*-GFP^low^/F4/80^int^/CD38^int^); and Q2/iii, (CD11b^high^/CD45^high^/*Cx3cr1*-GFP^high^/F4/80^high^/CD38^high^).

MG: Q3/i (CD11b^low^/CD45^low^/*Cx3cr1*-GFP^high^/F4/80^low^/CD38^low^); Q3/ii (CD11b^low^/CD45^high^/*Cx3cr1*-GFP^low^/F4/80^low^/CD38^high^) and Q3/iii (CD11b^high^/CD45^high^/*Cx3cr1*-GFP^high^/F4/80^high^/CD38^high^).

Taken together, and consistent with the IF staining results, these data show that BrM promotes TAM accumulation, more pronounced in the D122 model and to a lesser extent in Ret BrM, and that both TAM populations consist of heterogeneous populations corresponding to distinct activation states.

In *Ms4a3*^*cre*^*:R26*^*tdT*^*:Cx3cr1*^*Gfp*^ mice, tdT also labels neutrophils [44]. Since neutrophils can infiltrate BrM [20, 48, 49], we investigated their presence in the models used in this study. Neutrophils were identified by flow cytometry as CD11b⁺CD45⁺tdT⁺Cx3cr1-GFP⁻Ly6C⁺Ly6G⁺ cells in D122- and Ret-BrM-bearing *Ms4a3*^*cre*^*:R26*^*tdT*^*:Cx3cr1*^*Gfp*^ mice (Q1; S5A–D). As expected, these cells dominated the blood myeloid compartment (~64%), but neutrophils were rare in sham and BrM-bearing brains (4.8% in sham, 0.3% in D122, 3% in Ret; Fig. S5E). Thus, neutrophils represent a minimal fraction of the TME in these BrM models, validating the use of *Ms4a3*^*cre*^*:R26*^*tdT*^*:Cx3cr1*^*Gfp*^ mice for detecting MDM.

### Spatial organization of astrocytes, TAM, and blood vessels in parenchymal BrM

**Fig. 8:**
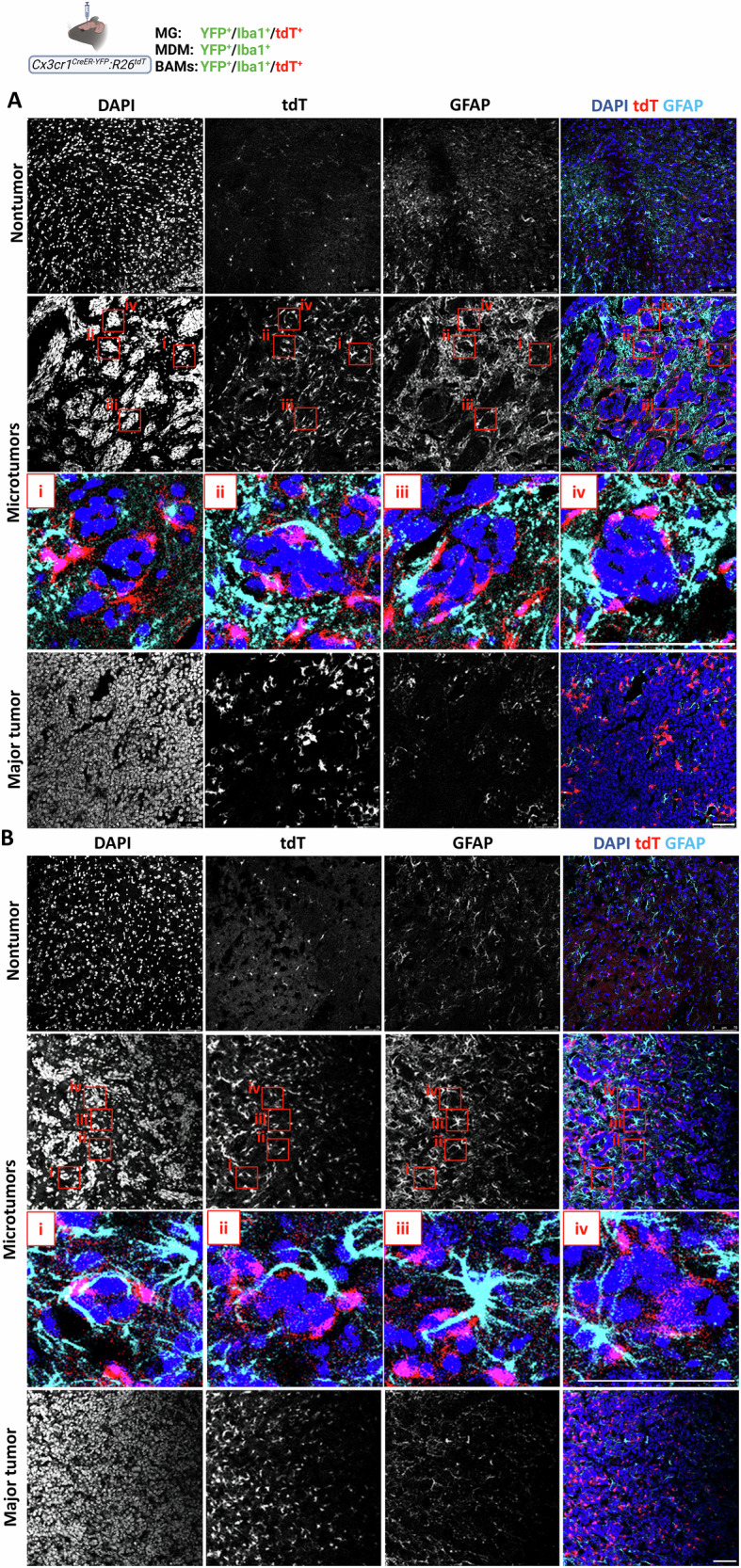
Spatial distribution of astrocytes in parenchymal BrM. *Cx3cr1*^*CreER-YFP*^*:R26*^*tdT*^ mice were intracranially injected with D122 (**A**) or EO771 (**B**) cells; sagittal sections were stained with DAPI and anti-GFAP. Representative confocal IF images showing DAPI, tdT, and GFAP signals. Individual channels and corresponding merged images are presented (*n* = 6–8). Boxed regions illustrate examples of astrocytes that either enwrap or do not contact microtumors, and their interactions with TA-MG. Note the activated morphology of astrocytes associated with microtumors, characterized by thickened processes and reduced branching, and their degenerative morphology within major tumors. Scale bar = 75 μm.

Previous studies have shown that astrocytes can regulate BrM growth [27, 50], in part through crosstalk with TAM and tumor cells [51, 52]. We therefore examined the spatial relationship between astrocytes, TA-MG, MDM, in parenchymal D122 and EO771 microtumors in *Cx3cr1*^*CreER-YFP*^*:R26*^*tdT*^ mice. As shown in Fig. 8A, B, reactive astrocytes identified by high GFAP expression and hypertrophic processes accumulated in the peritumoral regions of all three microtumor subgroups in both cancer types, similar to TA-MG. However, whereas TA-MG localized mainly to the tumor perimeter, astrocytes displayed a more dispersed distribution around the microtumors, with some also aligning along the tumor surface. Punctate GFAP signals detected inside major tumors (Fig. 8A, B), likely represented astrocytic debris from cells that had surrounded microtumors prior to their coalescence. Notably, TA-MG and reactive astrocytes intermingled in the peritumoral region of both cancer types and frequently appeared to interact (Fig. 8A, B). Because MDMs are also present at the microtumor periphery and in the intertumoral regions, particularly in the EO771 model (Fig. 4B, D), we further assessed the spatial relationship among astrocytes, TA-MG, and MDM in EO771 BrM using *Ms4a3*^*cre*^*:R26*^*tdT*^*:Cx3cr1*^*Gfp*^ mice, which enable more specific identification of MDM. In this model, all three cell types were observed intermingling at the tumor periphery, as well as between microtumors, where instances of direct interactions among them were also detected (Fig. S6).

To further characterize the properties of the parenchymal BrM lesions, we examined blood vessel presence and morphology using CD31 immunofluorescence and H&E staining. Many D122 and EO771 microtumors exhibited a perivascular pattern, with tumor cells wrapping blood vessels (Fig. S7). Both D122 and EO771 major tumors were highly vascularized, and their vasculature, as well as that of microtumors (albeit to a lesser extent), displayed enlarged lumina compared with blood vessels in nontumor regions (Fig. S7).

Notably, D122 and EO771 microtumors obtained by intracardiac injection also had a perivascular appearance and, moreover, were wrapped by TA-MG (Fig. S8). Thus, the intracranial BrM model recapitulates at least some features of BrM established by the intracardiac injection model.

### TAMs in ventricular BrM

**Fig. 9:**
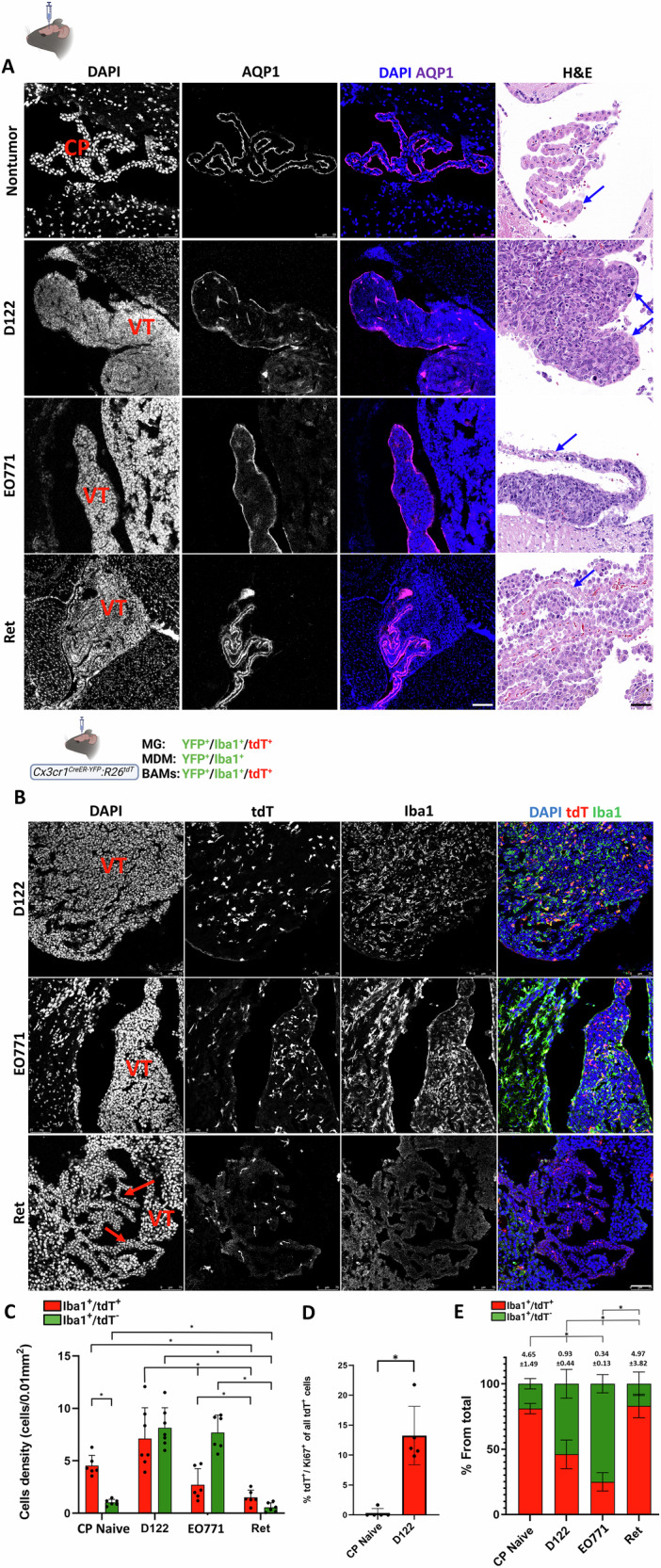
Characterization of ventricular BrM and their macrophage composition across different cancer types. *Cx3cr1*^*CreER-YFP*^*:R26*^*tdT*^ mice were intracranially injected with D122, EO771, or Ret cells. **A** IF staining with DAPI and AQP1 (shown as individual channels and merged images) or H&E indicates that D122 and EO771 lesions are located within the CP, whereas Ret lesions localize outside the CP. Blue arrows in H&E images indicate the CP epithelial layer. Scale bar, 50 μm. **B**, **C** Abundant tdT⁺ cells and MDM in D122 and EO771 ventricular BrM, but not in Ret. **B** Representative IF images of DAPI, tdT, and Iba1 in ventricular tumors (VT), shown as individual channels and merged images. Red arrows indicate the CP in Ret. Scale bar, 75 μm. **C** Quantification of tdT⁺ and MDM densities in naïve CP and the indicated ventricular BrM, expressed as cells/0.01 mm² (mean ± SEM; *n* = 6 mice for naïve, E0771, and Ret, and *n* = 7 for D122). Dots represent individual mice. ^*^q < 0.05. **D** Proliferation of tdT⁺ cells in naïve CP and D122 BrM-containing CP. Sagittal sections of naïve and D122 BrM-bearing mice were stained with anti-Ki-67 antibodies (see Fig. S9), and the percentage of tdT⁺ cells co-staining with Ki-67 was calculated. Values are presented as mean ± SEM (*n* = 5). ^*^q < 0.05. **E** Relative fractions of tdT^+^ cells and MDM from total Iba1^+^ cells in naïve CP and ventricular BrM. Subdivided bar graphs show mean ± SEM (*n* = 6 mice for naïve, E0771, and Ret, and *n* = 7 for D122). tdT^+^/Iba1^+^/MDM ratios shown above bars; statistics performed on ratio values. ^*^q < 0.05.

In addition to parenchymal lesions, intracranial implantation of all three cancer types also gave rise to ventricular BrM (Fig. 1), though lesion locations varied by tumor origin. D122 and EO771 lesions developed within the CP, between its epithelial sheaths as defined by AQP1 and H&E staining (Fig. 9A), whereas Ret lesions were exclusively located outside the CP (Fig. 9A).

Analysis of macrophage distribution in *Cx3cr1*^*CreER-YFP*^*:R26*^*tdT*^ mice showed that D122 and EO771 ventricular tumors contained abundant tdT⁺/Iba1⁺ cells and Iba1-only MDM, interspersed throughout the lesions (Fig. 9B, upper and middle). By contrast, Ret tumors contained very few tdT⁺/Iba1⁺ cells and MDM, primarily confined to the non-tumor CP, with only rare cells in the extra-CP regions (Fig. 9B, lower). Quantification revealed the highest tdT⁺/Iba1⁺ cell density in D122, the lowest in Ret, and comparable MDM densities in D122 and EO771, both markedly higher than in Ret tumors (Fig. 9C). Notably, while MDM densities were similar between D122 and EO771, tdT⁺/Iba1⁺ density was lower in EO771 (Fig. 9C), resulting in a higher relative contribution of tdT⁺/Iba1⁺ cells in D122 lesions (Fig. 9E). Assuming that cells within ventricular lesions may originate from the CP, we also quantified their density in naïve CP for comparison. Both tdT⁺/Iba1⁺ cells and, to a lesser extent, MDM were detected (Fig. 9C). MDM density was markedly higher in D122 and EO771 lesions than in naïve CP, indicating robust MDM infiltration into these tumors. By contrast, tdT⁺/Iba1⁺ cell density was comparable to that in naïve CP; however, given the much larger size of CP-containing tumors, this nevertheless implies expansion of this population, consistent with their increased proliferation (Ki-67 expression) in CP-associated lesions relative to naïve CP (Fig. 9D and S9). In Ret ventricular tumors, both tdT⁺/Iba1⁺ and MDM densities were even lower than in naïve CP, likely because these cells remained largely confined to the CP, which represents only a small fraction of the ventricular space. Indeed, tdT⁺/Iba1⁺/MDM ratios in Ret tumors resembled those in naïve CP (Fig. 9E), supporting the notion that most of these cells were restricted to the non-tumor CP region. The very low abundance of tdT⁺/Iba1⁺ cells and MDM within the extra-CP Ret ventricular lesions implies minimal involvement of these macrophage subsets in this BrM type.

**Fig. 10:**
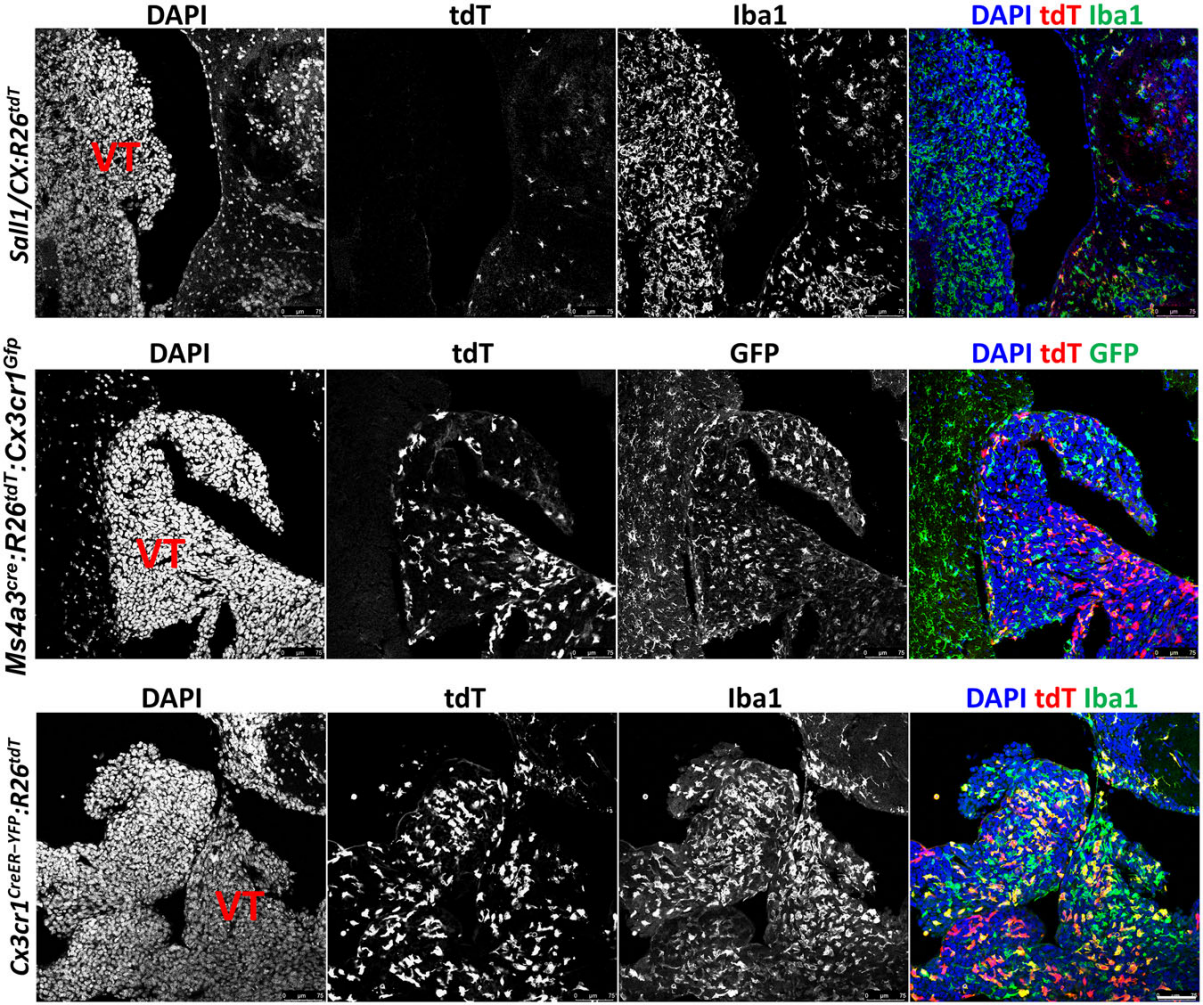
*Cx3cr1*-expressing cells in D122 ventricular BrM are not MG. *Sall1/CX:R26*^*tdT*^ *Ms4a3*^*cre*^*:R26*^*tdT*^*:Cx3cr1*^*Gfp*^ and *Cx3cr1*^*CreER-YFP*^*:R26*^*tdT*^ mice were intracranially injected with D122 cells. Sagittal sections were stained with DAPI and anti-Iba1 or anti-GFP antibodies. Representative confocal IF images (*n* = 4–6 mice) show DAPI, tdT, and Iba1 or GFP signals as individual channels and merged images, shown for ventricular tumor (VT) regions in the indicated lineage-tracing mouse models. In *Sall1/CX:R26*^*tdT*^ mice (upper panel), tdT labels only MG. Iba1⁺/tdT⁻ cells in these mice correspond to MDM. In *Ms4a3*^*cre*^*:R26*^*tdT*^*:Cx3cr1*^*Gfp*^ mice (middle panel), tdT labels MDM, whereas GFP labels MG and BAM. In *Cx3cr1*^*CreER-YFP*^*:R26*^*tdT*^ mice (lower panel), tdT labels MG and BAM. Scale bar: 75 μm.

Marker analysis in *Cx3cr1*^*CreER-YFP*^*:R26*^*tdT*^ revealed that most tdT⁺ cells in D122 and EO771 ventricular tumors expressed CD68 and F4/80, along with CD68⁺/tdT⁻ and F4/80⁺/tdT⁻ cells, likely representing MDM (Fig. S10A, B). However, tdT⁺ cells in D122 ventricular tumors lacked expression of the MG marker TMEM119, unlike surrounding parenchymal MG (Fig. S10C), raising the possibility that these tdT⁺ cells are not MG but another *Cx3cr1*-expressing population. Supporting this notion, *Sall1/CX:R26*^*tdT*^ mice, which specifically label MG with tdT via a splitCre approach [53], revealed numerous TAM (Iba1⁺ cells) within ventricular D122 tumors but only few, if any, tdT⁺ MG, despite MG abundance in the surrounding parenchyma (Fig. 10, upper). The presence of both MDM and non-MDM, *Cx3cr1*-expressing cells in ventricular BrM was further confirmed in *Ms4a3*^*cre*^*:R26*^*tdT*^*:Cx3cr1*^*Gfp*^ mice, in which monocyte-derived cells are fate-mapped (Fig. 10, middle). Consistent with the findings in *Cx3cr1*^*CreER-YFP*^*:R26*^*tdT*^ mice (Fig. 10, lower), ventricular D122 lesions in *Ms4a3*^*cre*^*:R26*^*tdT*^*:Cx3cr1*^*Gfp*^ animals contained both MDM (tdT⁺) and non-MDM *Cx3cr1*-expressing cells (tdT⁻/*Cx3cr1*-GFP⁺).

**Fig. 11:**
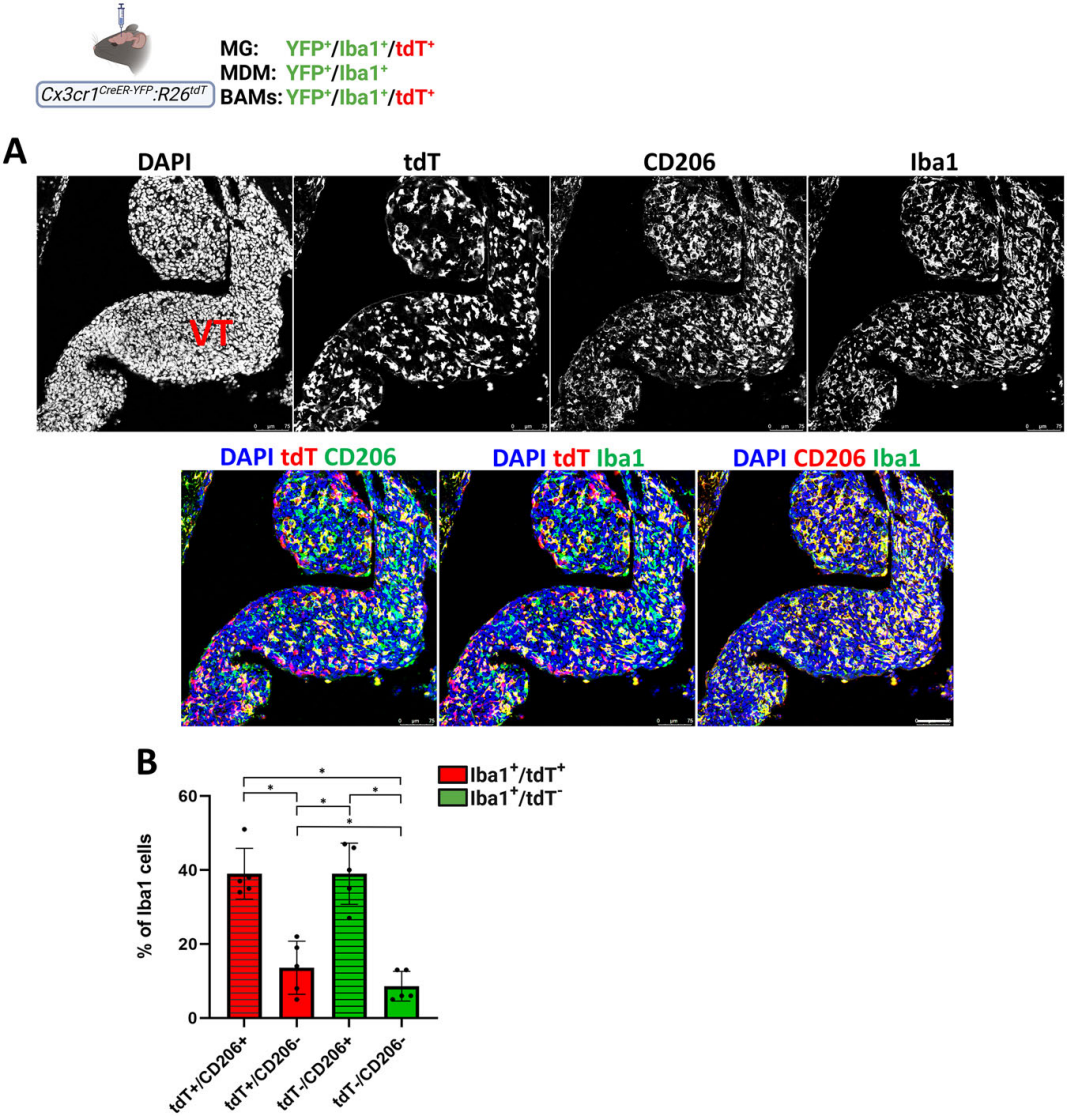
CD206 expression in macrophages within D122 ventricular BrM. *Cx3cr1*^*CreER-YFP*^*:R26*^*tdT*^ mice were intracranially injected with D122 cells. Sagittal sections were stained with DAPI, anti-CD206, and anti-Iba1 antibodies. **A** Representative confocal IF image of a ventricular (VT) BrM tumor mass. Upper panels show individual channels for the indicated signals, and lower panels display merged images of the same field (*n* = 5). Scale bar: 75 μm. **B** Quantification of the percentage of tdT⁺/Iba1⁺ (red) and tdT⁻/Iba1⁺ (green) ventricular macrophages that express (striped bars) or do not express (solid bars) CD206 from total Iba1⁺ cells. tdT⁺/Iba1⁺ and tdT⁻/Iba1⁺ cells likely correspond to BAM and MDM, respectively. Data are presented as mean ± SEM (*n* = 5). ^*^q < 0.05.

We hypothesized that these non-MDM *Cx3cr1*^*+*^ cells represent CP-BAM, which are labelled in both *Cx3cr1*^*CreER-YFP*^*:R26*^*tdT*^ and *Ms4a3*^*cre*^*:R26*^*tdT*^*:Cx3cr1*^*Gfp*^ lineage models due to *Cx3cr1* expression [29]. To test this, we assessed expression of the BAM marker CD206 [54] in *Cx3cr1*^*CreER-YFP*^*:R26*^*tdT*^ mice bearing D122 BrM. The majority of tdT⁺/Iba1⁺ cells (75%) in ventricular CP-BrM co-expressed CD206, supporting their classification as CP-BAM (Fig. 11A, B). Notably, CD206 positivity alone cannot uniquely identify BAMs; indeed, most MDMs in CP-BrM (78%) also expressed CD206. Importantly, ventricular tumors generated by intracardiac injection of D122 cells in *Cx3cr1*^*CreER-YFP*^*:R26*^*tdT*^ mice also contained abundant MDM and tdT⁺/Iba1⁺ cells (Fig. S11), suggesting that the presence of these TAM populations within ventricular BrM is a general feature, independent of the method used for their generation. Taken together, these results indicate that CP-BAM, together with MDM, constitute the predominant TAM compartment in ventricular CP-BrM.

### TAMs in leptomeningeal BrM

**Fig. 12:**
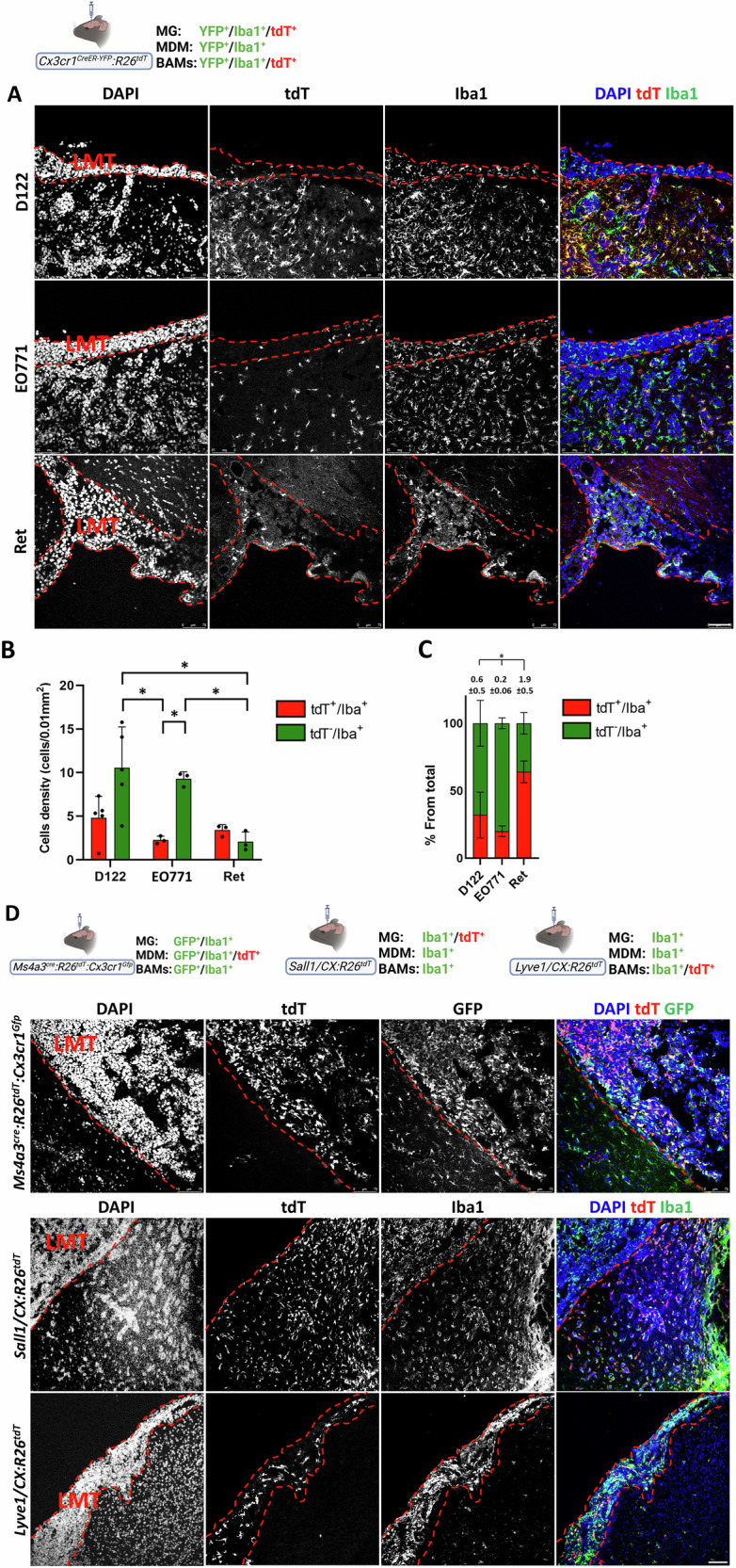
Macrophage composition in leptomeningeal BrM. *Cx3cr1*^*CreER-YFP*^*:R26*^*tdT*^ mice were intracranially injected with D122, EO771, or Ret cells. Sagittal sections were stained with DAPI and anti-Iba1 antibodies. **A** Representative confocal IF image showing DAPI, tdT, and Iba1 signals as individual channels and merged images in leptomeningeal tumors (LMT) (*n* = 4–5 mice). Dashed lines outline tumor margins. Scale bar, 75 μm. **B** Quantification of tdT^+^/Iba1^+^ (red) and tdT^−^/Iba1^+^ (green) cells from total Iba1^+^ (mean ± SEM; *n* = 5 for D122 and *n* = 3 for EO771 and Ret). ^*^q < 0.05. **C** Relative fractions of tdT^+^/Iba1^+^ cells and MDM from total Iba1^+^ cells in leptomeningeal BrM. Subdivided bar graphs show mean ± SEM (*n* = 5). tdT^+^/Iba1^+^/MDM ratios shown above bars; statistics performed on ratio values. *q < 0.05. **D** Lineage tracing shows the absence of MG and the presence of BAM in the leptomeningeal BrM. *Ms4a3*^*cre*^*:R26*^*tdT*^*:Cx3cr1*^*Gfp*^*, Sall1/CX:R26*^*tdT*^ and *Lyve1/CX:R26*^*tdT*^ mice were intracranially injected with D122 cells. Sagittal sections were stained with DAPI, anti-GFP, and anti-Iba1 antibodies. Representative confocal IF images showing DAPI, tdT, and GFP signals as individual channels and merged images (scale bar: 75 μm; *n* = 3). Dashed lines delineate the borders of leptomeningeal lesions. Notably, tdT signal (MG) is absent from leptomeningeal BrM in *Sall1/CX:R26*^*tdT*^ mice, whereas tdT signal (BAM) is present in leptomeningeal BrM in *Lyve1/CX:R26*^*tdT*^ mice.

Next, we analyzed the TAM composition in leptomeningeal BrM using the *Cx3cr1*^*CreER-YFP*^*:R26*^*tdT*^ model. Leptomeningeal BrM of all three cancer types contained *Cx3cr1-*expressing tdT⁺/Iba1⁺ cells at comparable densities (Fig. 12A, B). Iba1-only MDM were also detected, but their density was lower in Ret BrM compared to D122 and EO771 lesions (Fig. 12A, B). Consequently, the relative contribution of tdT⁺/Iba1⁺ cells to the total TAM pool (tdT⁺/Iba1⁺/MDM ratio) was higher in Ret BrM than in D122 or EO771 BrM (Fig. 12C). Analysis of TAM composition in leptomeningeal BrM-bearing *Ms4a3*^*cre*^*:R26*^*tdT*^*:Cx3cr1*^*Gfp*^ mice confirmed the results obtained with *Cx3cr1*^*CreER-YFP*^*:R26*^*tdT*^ mice, showing that leptomeningeal D122 BrM contained *Cx3cr1*-expressing GFP-only cells, as well as tdT⁺ MDM (Fig. 12D, upper). To determine the identity of the *Cx3cr1*-expressing cells in leptomeningeal BrM, we assessed TMEM119, CD68, and F4/80 expression in D122-BrM-bearing *Cx3cr1*^*CreER-YFP*^*:R26*^*tdT*^ mice. The results showed that the tdT^+^ cells did not express the microglial marker TMEM119, but expressed Iba1, CD68, and F4/80 (Fig. S12), suggesting that, as above, they are not MG. Again, this conclusion was supported by analysis of *Sall1/CX:R26*^*tdT*^ mice, in which their leptomeningeal D122 BrM contained many TAM (Iba1^+^ cells), but not MG (tdT^+^/Iba1^+^ cells), even though MG were abundant in the adjacent parenchyma, particularly as expected, around parenchymal microtumors (Fig. 12D, middle).

The leptomeninges contain a distinct BAM population that expresses Lyve1, as well as *Cx3cr1* [29, 55], and these cells can be detected using the *Cx3cr1*^*CreER*^ system [29]. We therefore speculated that the *Cx3cr1*-expressing cells observed in leptomeningeal BrM established in *Cx3cr1*^*CreER-YFP*^*:R26*^*tdT*^ and *Ms4a3*^*cre*^*:R26*^*tdT*^*:Cx3cr1*^*Gfp*^ mice represent leptomeningeal BAM. To test this, we used *Lyve1/CX:R26*^*tdT*^ splitCre mice, in which leptomeningeal BAM are specifically labeled by tdT [53]. Indeed, leptomeningeal D122 BrM in these mice contained a substantial number of tdT⁺ BAM, in addition to MDM (Iba1-only) (Fig. 12D, lower). Collectively, these results demonstrate that the TAM population in leptomeningeal BrM is composed of Lyve1⁺ BAM and MDM.

## Discussion

Therapeutic options for BrM remain limited. Given the central role of TAM in regulating BrM progression, these cells represent attractive therapeutic targets. A major challenge, however, is the need for selective targeting of distinct TAM subpopulations, whose roles may vary depending on the primary tumor of origin, the brain compartment affected, and the stage of metastatic growth.

To date, the spatial organization and diversity of TAM subpopulations across different BrM models have not been systematically characterized. In this study, we used four complementary, independent lineage-tracing mouse systems that together enabled us to define the spatial distribution, relative abundance, and phenotypic features of MG, MDM, and BAM in parenchymal, ventricular, and leptomeningeal BrM derived from lung, breast, and melanoma cells. Identity assignments and distribution patterns for the different macrophage types were supported by concordant results across the lineage-tracing systems and by consistent marker profiles (TMEM119, CD68, F4/80, CD206). Moreover, the use of multiple models provided cross-validation of our findings across diverse experimental contexts.

Our analyses revealed distinct compartmental colonization patterns across the three BrM models used in this study. D122 and EO771 BrM were abundant in the parenchyma, ventricles, and leptomeninges, whereas Ret melanoma BrM preferentially developed in the ventricles and leptomeninges, with only rare occurrence in the parenchyma. AQP1/H&E anatomy further showed that D122 and EO771 ventricular lesions were largely confined to the CP, whereas Ret melanoma lesions predominantly arose in the interventricular space, outside the CP. These findings highlight that BrM distribution and architecture are shaped by both tumor type and brain niche

### Distribution of MG, MDM, and astrocytes in parenchymal BrM

D122 and EO771 parenchymal BrM exhibited a broad size range. To assess how TAM composition relates to tumor growth, we stratified lesions into microtumors, further classified as small, medium, or large, and major tumors, which likely arise from the coalescence of multiple microtumors. These subgroups probably represent progressive expansion stages. Most microtumors contained blood vessels and exhibited a perivascular architecture consistent with tumor expansion by blood vessel co-option [56]. Thus, we cannot exclude the possibility that some microtumors were interconnected.

TA-MG showed predominant accumulation at the periphery of D122 and EO771 microtumors. Proliferation, as evidenced by Ki-67 expression, appeared to underlie this accumulation, although recruitment of migrating MG from more distal regions cannot be ruled out. Some proliferating TA-MG were not in direct tumor contact, suggesting that soluble cues, in addition to physical interaction, may drive their expansion.

Although MDMs were detectable within microtumors, their frequency was much lower than that of TA-MG, and they were largely absent from the smallest lesions, particularly in D122 BrM. This pattern, together with the pronounced accumulation and proliferation of TA-MG around small microtumors, suggests that MG constitutes the earliest TAM responders to parenchymal BrM, with MDM infiltration occurring later as the lesions expand. Consequently, TA-MG may play a dominant role during the initial stages of BrM outgrowth, whereas MDM likely assumes greater importance at later stages, when their numbers eventually surpass those of TA-MG. The increased MDM content observed in larger tumors, together with their minimal proliferation (Fig. S3), is consistent with monocyte influx into the lesions, as previously suggested [24]. Such recruitment may be facilitated, at least in part, by the abnormal morphology of blood vessels within parenchymal BrM, which could enhance vascular permeability and monocyte entry.

Our findings, demonstrating MG predominance at the microtumor margin and size-dependent alterations in TAM composition, are consistent with previous studies [19, 24, 56–58]. Taken together, our findings indicate that microtumors and major tumors differ markedly in MG and MDM composition and spatial distribution, whereas the three microtumor subgroups display only subtle variations.

Beyond their expansion, MG associated with parenchymal BrM also undergo phenotypic reprogramming, acquiring MDM-like features as reflected by the downregulation of the homeostatic marker TMEM119 and upregulation of macrophage-associated proteins CD68 and F4/80. Similar shifts in MG identity have been reported in glioma [43, 59] and in human BrM [20], suggesting that such transitions may represent a common response of MG to brain malignancies. Flow cytometry further revealed that both MG and MDM populations in BrM-bearing brains are heterogeneous, distinguished by varying expression of CD11b, CD45, F4/80, CD38, and *Cx3cr1*-GFP, pointing to functional diversity within these macrophage subsets. The mechanisms through which TA-MG and MDM influence BrM progression and how these functions relate to their spatial distribution remain to be fully elucidated. Nevertheless, our observations of cell engulfment and CD68 expression suggest a role for phagocytosis. This is consistent with prior work showing that MG phagocytic activity can modulate brain tumor growth [51, 58, 60, 61].

Of note, although the *Cx3cr1* reporter labels MG, it can also detect perivascular BAM. Therefore, we cannot fully exclude the possibility that some cells we identified as MG are perivascular BAMs. Nonetheless, perivascular BAM are present only along a subset of parenchymal blood vessels, and were suggested to be absent from capillaries [62, 63]. Moreover, the spatial distribution of TA-MG around perivascular tumors in *Sall1/CX:R26*^*tdT*^ mice closely resembled that observed in *Cx3cr1* reporter mice. We therefore concluded that the majority of *Cx3cr1*-expressing cells detected in the parenchyma are most likely MG.

Astrocytes also accumulated around D122 and EO771 microtumors, consistent with earlier work [56]. However, their organization differed from that of TA-MG: while MG closely wrapped the tumor surface, astrocytes displayed a more diffuse distribution. We also noted the presence of monocyte-derived cells at the borders and in the spaces between microtumors, an effect more pronounced in EO771 than in D122 BrM. These cells are likely MDM, though we cannot exclude the possibility that some monocyte-derived cells acquired MG-like features, as reported in aging [46]. The shared tendency of MG, astrocytes, and MDM to cluster at the microtumor surface highlights this interface as a key niche where TME cells may influence early tumor expansion. The co-localization of MG, MDM, and astrocytes at the microtumor interface further raises the possibility of active crosstalk among these cell types and tumor cells in shaping BrM progression. As lesions enlarge, MG and MDM also infiltrate the tumor interior, suggesting a dynamic reorganization of the TME landscape.

### TAM content in leptomeningeal and ventricular BrM

Our results using the *Sall1/CX:R26*^*tdT*^ mice and an MG marker did not detect MG in leptomeningeal and ventricular BrM. In contrast, the findings indicate that BAMs are a component of the TAM population in leptomeningeal and CP-derived BrM, and that they expand in response to tumor growth. This conclusion is supported by the presence of *Cx3cr1*-expressing, non-MDM/non-MG cells within these BrM that expressed macrophage markers (CD68, F4/80) using both the *Cx3cr1*^*CreER-YFP*^*:R26*^*tdT*^ and *Ms4a3*^*cre*^*:R26*^*tdT*^*:Cx3cr1*^*Gfp*^ systems, an approach previously applied for BAM identification [29, 64–67]. This was further corroborated by *Lyve1/CX:R26*^*tdT*^ mice [53], which specifically trace meningeal and perivascular BAM, confirming their presence in leptomeningeal BrM. In CP-BrM, BAM identity was additionally supported by expression of CD206, a known BAM marker.

The presence of BAM across BrM derived from different cancer types underscores their potential role in regulating tumor niches at the brain borders. This is consistent with recent work showing that leptomeningeal BAM fosters breast cancer growth in the meninges [35] and that dural BAM creates immunosuppressive environments that restrain T cell activity [68]. Whether CP-BAM similarly shapes the ventricular BrM niche remains an open question and warrants further study.

In addition to resident BAM, we also observed monocyte-derived cells in both CP-BrM and leptomeningeal BrM, where they were more abundant than BAM. These cells are most likely classical MDM, though we cannot exclude the possibility that some infiltrating monocytes adopt BAM-like properties, particularly in CP-BRM, because BAM replacement by bone marrow–derived cells has been reported in CP [29, 64]. Collectively, our results show that BAM and MDM comprise the TAM populations at the brain borders of BrM, supporting the notion that both cell types play functional roles in these regions. Moreover, our findings reinforce a compartmentalized model of TAM biology in BrM: infiltrating MDM promote tumor growth across brain compartments, whereas resident macrophages - MG in the parenchyma and BAM at the borders - act within their native microenvironments.

### Effect of primary tumor type on TAM composition

Comparing TAM composition, proportional abundance, and distribution patterns across BrM derived from different primary cancers revealed both commonalities and tumor type–specific differences. Overall, D122 and EO771 BrM elicited broadly similar TAM responses: in parenchymal lesions, both tumor types showed comparable distribution patterns of TA-MG and MDM across all BrM subgroups, and in both cases the relative abundance of MDM increased as tumors grew. However, subtle differences were evident. EO771 BrM contained higher MDM proportions within the TAM pool and greater MDM infiltration in microtumors than D122 BrM. Conversely, D122 BrM exhibited higher densities of ventricular BAM in CP-BrM. This suggests that EO771 cells may be more efficient at recruiting MDM across brain compartments, whereas D122 cells preferentially recruit BAM in the CP-BrM.

Ret ventricular BrM displayed a markedly different ventricular location and TAM landscape. Unlike D122 and EO771, Ret lesions developed outside the CP in the intraventricular space and contained very few MDM or BAM, which were largely confined to the adjacent CP. Notably, in contrast to ventricular Ret BrM, Ret leptomeningeal BrM did contain MDM and leptomeningeal BAM, albeit at lower densities than observed in D122 or EO771 leptomeningeal lesions. Together, these findings suggest that Ret tumor cells are generally less efficient at recruiting MDM and that their capacity to engage BAM is brain compartment–dependent. These results highlight that TAM composition is shaped not only by tumor cell genetics, consistent with previous studies [23, 33, 34], but also by the anatomical location of the BrM. The underlying mechanisms may involve differences in stromal content and/or vascular architecture.

Our use of intracranial injection yielded BrM colonization patterns for D122 and EO771 (parenchyma, ventricles, leptomeninges) and Ret (ventricles, leptomeninges) that are consistent with prior reports using intracranial, intracardiac, or intracarotid approaches for D122 and EO771 [56, 58, 69–71], as well as patient-derived melanoma [72]. Moreover, the presence of perivascular-like parenchymal BrM, the accumulation of MG around these small lesions, and the appearance of BAM-like cells in ventricular D122 BrM were all reproduced in BrM established by intracardiac implantation. Collectively, this supports the notion that the intracranial metastatic model recapitulates at least some of the features of BrM generated by the intracardiac injection model and is therefore a suitable system for studying BrM.

## Conclusion

Our findings demonstrate that the composition and abundance of TAM subtypes in BrM are shaped not only by the primary cancer type but also by their spatial distribution relative to the lesion (adjacent, edge, or core) and the brain compartment in which the BrM arises (parenchyma, ventricles, or leptomeninges). Infiltrating MDM were consistently involved across all compartments, whereas resident macrophages contributed primarily within their native territories, MG in the parenchyma, and BAM at brain borders (Fig. S13). These findings highlight that successful TAM-targeted therapies must consider both the genetic origin of the primary tumor and the spatial distribution of TAM subsets within BrM and across distinct brain compartments. Accordingly, future investigations should aim to clarify the spatial roles of TAM subtypes in BrM and their reciprocal interactions with tumor cells and other components of the TME.

## Materials and methods

### Reagents

Unless otherwise stated, reagents were purchased from Sigma-Aldrich (St. Louis, MO) and cell culture media from Invitrogen Life Technologies (Paisley, UK).

### Illustration

Schematic mouse illustrations were generated using BioRender.com.

### Mice

Homozygote B6.129P2(Cg)-*Cx3cr1*tm2.1(cre/ERT2)Litt/WganJ (*Cx3cr1*:^CreER-IRES-EYFP^) (Jackson Laboratory) (RRID:IMSR_JAX:021160) mice were bred with homozygote B6.Cg-*Gt(ROSA)26Sor*^*tm9(CAG-tdTomato)Hze*^/J reporter mice (Jackson Laboratory) to yield *Cx3cr1*^*CreER-IRES-EYFP/+*^:*Rosa26*^*lsl-TdTomato/+*^ (herein *Cx*_*3*_*cr1*^*CreER-YFP*^*:R26*^*tdT*^) mice. *Sall1*^*ncre*^*:Cx3cr1*^*ccre*^: *Rosa26*^*lsl-TdTomato/TdTomato*^ (herein *Sall1/CX:R26*^*tdT*^) and *Lyve1*^*ncre*^*:Cx3cr1*^*ccre*^:*Rosa26*^*lsl-TdTomato/TdTomato*^ (herein *Lyve1/CX:R26*^*tdT*^) mice [53], as well as *Ms4a3*^*Cre/+*^*:R26*^*LSL-TdTomato/+*^*:Cx3cr1*^*Gfp/+*^ (herein *Ms4a3*^*cre*^*:R26*^*tdT*^*:Cx3cr1*^*Gfp*^) mice [73], are all on C57BL/6 background. Animals were bred and maintained at the Tel-Aviv University animal facility. Tumor-bearing mice were monitored three times per week for clinical signs of morbidity. Mice showing morbidity symptoms were euthanized. All animal studies were performed according to protocols approved by the Tel Aviv University Animal Care and Use Committee.

### Cell culture

Mouse D122 Lewis lung carcinoma (LLC) cells [40] were provided by Shamgar Ben-Eliyahu. Mouse Ret melanoma cells [42, 74] and mouse EO771 breast cancer cells [41] were provided by Neta Erez (all syngeneic to C57BL/6J mice). Cells were cultured in RPMI-1640 medium with L-glutamine (01-100-1A, Sartorius) supplemented with 10% fetal calf serum and 1% penicillin–streptomycin. EO771 cultures were additionally supplemented with 1% sodium pyruvate and 1% HEPES buffer. All cells were maintained at 37 °C in a humidified atmosphere with 5% CO₂.

### Intracranial injection

Three-month-old *Cx*_*3*_*cr1*^*CreER-YFP*^*:R26*^*tdT*^, *Sall1/CX:R26*^*tdT*^, *Lyve1/CX:R26*^*tdT*^ or *Ms4a3*^*cre*^*:R26*^*tdT*^*:Cx3cr1*^*Gfp*^ mice were anesthetized with isoflurane and positioned in a Kopf stereotaxic alignment system. A 1-cm midline incision was made in the scalp to expose the skull, and a 2-mm burr hole was drilled 1 mm posterior and 1.5 mm lateral to the bregma. Using a Hamilton 10-μL syringe fitted with a 31-gauge needle, 1 × 10⁴ D122 cells or 1 × 10³ Ret cells were injected into male or female mice, and 1 × 10⁴ EO771 cells were injected into female mice. Cells (3 μL in RPMI) were delivered 4 mm below the cortical surface at a rate of 1 μL/min. To minimize backflow, the needle was left in place for 1 min before being slowly withdrawn. The incision was sutured, and mice were allowed to recover in their home cages. Animals were sacrificed 21 days post-injection (dpi) for D122 and EO771 tumors, and 14 dpi for Ret tumors.

### Intracardiac injections

Three-month-old *Cx*_*3*_*cr1*^*CreER-YFP*^*:R26*^*tdT*^ mice were anesthetized with 10% ketamine/xylazine, and the fur was removed from the mouse thorax. Female mice were injected with 1 × 10⁵ EO771 cells, while both male and female mice were injected with 1 × 10⁵ D122 or Ret cells, suspended in 100 μL PBS. Injections were performed into the left ventricle of the heart using a 29-gauge needle under ultrasound guidance (Vevo 770 High-Resolution System; VisualSonics Inc.). Mice were sacrificed at 14 dpi for all three cell lines.

### Immunostaining

Mice were euthanized using excess CO₂ or sodium pentobarbital (200 mg/kg; i.p.) and perfused through the left ventricle with PBS. Brains were removed and fixed in 4% paraformaldehyde for 18 h at 4 °C, followed by incubation in 30% sucrose for 72 h. Sagittal sections (20 μm) were prepared from cryo-embedded tissue using a Leica CM1950 cryostat. Sections were blocked in PBS containing 0.3% Triton X-100, 1% BSA, and 5% donkey serum for 1 h at room temperature, and then incubated overnight at 4 °C with primary antibodies. After washing with PBS, sections were incubated with secondary antibodies for 1 h at room temperature, followed by PBS washes and counterstaining with DAPI (Sigma-Aldrich, cat. no. D9542, 10 min).

Primary antibodies: chicken polyclonal anti-GFP (1:1000, Abcam, cat. no. ab13970), rabbit monoclonal anti-Iba1 (1:400, Abcam, cat. no. ab178846), rabbit monoclonal anti-TMEM119 (1:800, Abcam, cat. no. ab209064), rat monoclonal anti-F4/80 (1:800, Abcam, cat. no. ab6640), rabbit polyclonal anti-GFAP (1:2000, Dako, cat. no. Z0334), rabbit polyclonal anti-Ki-67 (1:500, Abcam, cat. no. ab15580), rat monoclonal anti-CD31 (1:200, BD Biosciences, cat. no. 550274), rat monoclonal anti-CD68 (1:500, Bio-Rad, cat. no. MCA1957), rabbit monoclonal anti-AQP1 (1:500, Abcam, cat. no. ab300463), goat polyclonal anti-MMR/CD206 (1:200, R&D Systems, cat. no. AF2535).

Secondary antibodies: donkey anti-chicken IgG (H + L) Alexa Fluor® 488 (1:500, Jackson ImmunoResearch, cat. no. 703-545-155), donkey anti-rat IgG (H + L) Alexa Fluor® 647 (1:500, Jackson ImmunoResearch, cat. no. 712-607-003), donkey anti-rabbit IgG (H + L) Alexa Fluor® 647 (1:500, Thermo Fisher, cat. no. 712-607-003), donkey anti-goat IgG (H + L) Alexa Fluor® 647 (1:500, Thermo Fisher, cat. no. 705-605-003).

tdTomato fluorescence was detected directly, whereas YFP fluorescence was visualized by immunostaining with anti-GFP antibodies due to reduced native signal after fixation. Sections were mounted with Mowiol 4–88 homemade mounting medium and coverslipped. For histopathology, formalin-fixed, paraffin-embedded tumor-bearing brains were cut into 6 μm sagittal sections and stained with H&E.

Image acquisition: Whole-section H&E images were acquired using an Aperio Versa 200 (Leica Biosystems) slide scanner with a 20×/0.75 air objective, and whole-section fluorescent images were captured with an Olympus IX83 microscope equipped with a 20×/0.7 air objective. Confocal images were acquired on Leica SP8 microscopes with 20×/0.75 air or 63×/1.4 oil objectives at 1024 × 1024 pixel resolution. For phagocytosis assays, 12 μm Z-stacks were collected at 63× objective with 0.3–0.5 μm step size. Image processing and analysis were performed using ImageJ software.

### Quantification of the density of MG, MDM, and BAM

Parenchymal tumor regions were manually segmented in ImageJ based on DAPI staining. The resulting binary masks were then exported and analyzed in MATLAB (Version 2022a, MathWorks). Tumors were classified into four size categories according to their segmented area: small: 70–1300 pixels (130–738.6 µm²), medium: 2000–4000 pixels (1136–2272 µm²), large: 6000–10000 pixels (3409–6818 µm²), and major masses (>338,511.7 µm²). Because microtumor sizes form a continuum, the size ranges were defined to maximize separation between categories while minimizing overlap. The density of MG (tdTomato-positive [tdT⁺] or Iba1/GFP⁺/tdT⁻ cells) and MDM (Iba1-only or tdT⁺ cells) was determined in *Cx3cr1*^*CreER-YFP*^*:R26*^*tdT*^ or *Ms4a3*^*cre*^*:R26*^*tdT*^*:Cx3cr1*^*Gfp*^ mice, respectively. Two tumor-associated regions were analyzed in the categorized microtumors: (i) the tumor periphery, defined as a 7-pixel–wide band centered on the tumor border and extending equally inward toward the tumor core and outward into the surrounding tissue; and (ii) the tumor interior, defined as the remaining tumor area after excluding the periphery. MG and MDM cell densities were quantified for each microtumor subgroup in both the interior and periphery, as well as across the entire tumor area (interior + periphery) and in major tumor masses. To quantify MDM density between microtumors in *Ms4a3*^*cre*^*:R26*^*tdT*^*:Cx3cr1*^*Gfp*^ mice, MDM located ≥4 µm from any microtumor were counted, and their density was calculated relative to the tumor-free area in each image. The 4-µm threshold was selected to exclude cells potentially at the tumor border. The tumor-free area was determined by subtracting the combined area of all tumor regions, identified in ImageJ as DAPI-dense regions larger than 130 µm², from the total imaged area (338,511.7 µm²). The density of leptomeningeal and CP BAM in *Cx3cr1*^*CreER-YFP*^*:R26*^*tdT*^ mice was calculated as the number of tdT⁺ cells within the leptomeninges or CP, normalized to the tumor area or, in naïve brains, to the CP area. A workflow of these quantification analyses is shown in Fig. S1.

### Quantification of proliferating MG and CP BAM

Proliferating MG or BAM in the parenchyma or CP of *Cx3cr1*^*CreER-YFP*^*:R26*^*tdT*^ mice, respectively, were defined as tdT^+^ cells that co-express Ki-67 (tdT^+^/Ki-67^+^). Proliferating and non-proliferating MG were counted in images captured from BrM-containing brain sections in nontumor regions, in the different microtumor subgroups, and in the major tumor. To determine the percentage of proliferating MG that are in direct (touching) or indirect (non-touching) contact with the different microtumor subgroups, touching proliferating MG were defined as proliferating MG that contacted the tumor edge or were present inside the tumor mass. Non-touching proliferating MG were defined as MG located at ≥4 µm from the tumor edge. CP-associated BAM (CP-BAM) were defined as tdT^+^ cells present within the CP of naïve mice or within CP-BrM. Images of CP- or CP-BrM–containing brain sections from naïve mice and from D122 BrM-bearing mice were acquired, and tdT⁺, as well as tdT⁺/Ki-67⁺ cells within these structures, were quantified. To analyze proliferating BAM in Ret BrM, images were obtained from BrM-containing ventricles, and tdT⁺ and tdT⁺/Ki-67⁺ cells were counted across the entire ventricular space, including both tumor mass and CP. The frequency of proliferating MG and BAM was expressed as the percentage of tdT⁺/Ki-67⁺ cells relative to the total tdT⁺ population

### Flow cytometry

Mice were anesthetized with CO2 and perfused with PBS, and their brains were removed. Brains from sham- or tumor cell-injected mice were mechanically minced and incubated with collagenase type 2 (1 mg/mL, Worthington Biochemical Corporation) and Dispase II (2 mg/mL, Roche) for 20 min, followed by 20 min incubation with DNase I (6 U/1 mL, Thermo Fisher Scientific). The homogenates were washed with RPMI with 20% fetal calf serum (Biological Industries) and then with serum-free RPMI and filtered through a 70-μm mesh. The resulting infiltrate was centrifuged for 10 min at 4 °C at 500 g. The pellet was suspended in 5 mL of 90% Percoll solution and centrifuged at 800 g at 4 °C for 25 min, w/o acceleration or braking. The cell pellet was resuspended in PBS supplemented with 1% EDTA and 1% fetal bovine serum (GibcoTM), followed by treatment with red blood cell lysis buffer (BD Biosciences) and incubation with antibodies for 45 min on ice. For blood analysis, blood was collected from the facial vein under isoflurane anesthesia, treated with red blood cell lysis buffer, washed, resuspended in flow cytometry buffer, and incubated with antibodies. The following antibodies were used: anti-Ly6C (Clone HK1.4, Biolegend, #128011), anti-Ly6G (Clone A18, Biolegend, #BLG-127613), anti-CD45 (Clone 30-F11, Biolegend, #103116), anti-CD11b (Clone M1/70, Biolegend, #101215), anti-CX3CR1 (Clone SA011F11, Biolegend, cat.no.149007), anti-CD38 (Clone-90, Biolegend, #102711) and anti-F4/80 (Clone-BM8, Biolegend, #123115). DAPI (0.2 μg/mL) was used to exclude dead cells. Analysis was performed, and data were acquired on a Cytoflex 4 L Flow Cytometer. Postacquisition analysis was performed with FlowJo Software (Tree Star, FlowJo LLC; Ashland, Oregon, version X.10 RRID:SCR_008520).

### Statistical analysis

The data were analyzed using three-way ANOVA [Figs. 2D, 3B (black), and 4B (black)], two-way ANOVA (Figs. 2B, 3C, 4C, 9C, and 12B), one-way ANOVA [Figs. 2C, 3B (blue), D, 4B (blue), D, S4E, 5B, C, 9E, 11B, and 12C] or Two-tailed Mann–Whitney *U*-test (Fig. 9D) after squared root [Figs. 3B (blue), 4B (blue), S4E, 5B, C, and 11B], cube root [Figs. 2D, 3B (black), and 4B (black)] or log transformation (Figs. 2B, C, 3C, 4C, D, 9C, E, and 12B, C) for normal distribution, followed by post-hoc linear contrasts comparing the individual experimental groups, and corrected for multiple comparisons using the False Discovery Rate method (two-stage FDR method of Benjamini, Krieger, and Yekutieli [75]. The correlation between tumor size and cell density (Fig. 2B) was assessed using Spearman’s correlation between tumor size rank and cell density within each experimental group. Values of *q* < 0.05 were considered statistically significant. Data are expressed as mean values ± SEM.

### Ethics declarations

All studies were conducted in accordance with protocols approved by the Tel Aviv University Animal Care and Use Committee under protocol number TAU-LS-IL-2309-155-5.

## Supplementary information

Supplementary Figures

Supplementary Figure Legends

## Data Availability

Data will be made available on reasonable request.

## Code availability

The custom code/scripts used in this study are available from the corresponding author upon reasonable request.
