# Supplementary material for: Brain metastases exhibit distinct spatial patterns of resident and infiltrating macrophages
Source: Cell Death Discov. 2026 Apr 1;12:211. doi: 10.1038/s41420-026-03084-0 (PMC13168416; doi:10.1038/s41420-026-03084-0)
Supplement: Supplementary file 1 — Supplementary Figures [file 41420_2026_3084_MOESM1_ESM.pdf]

Fig. S1

A

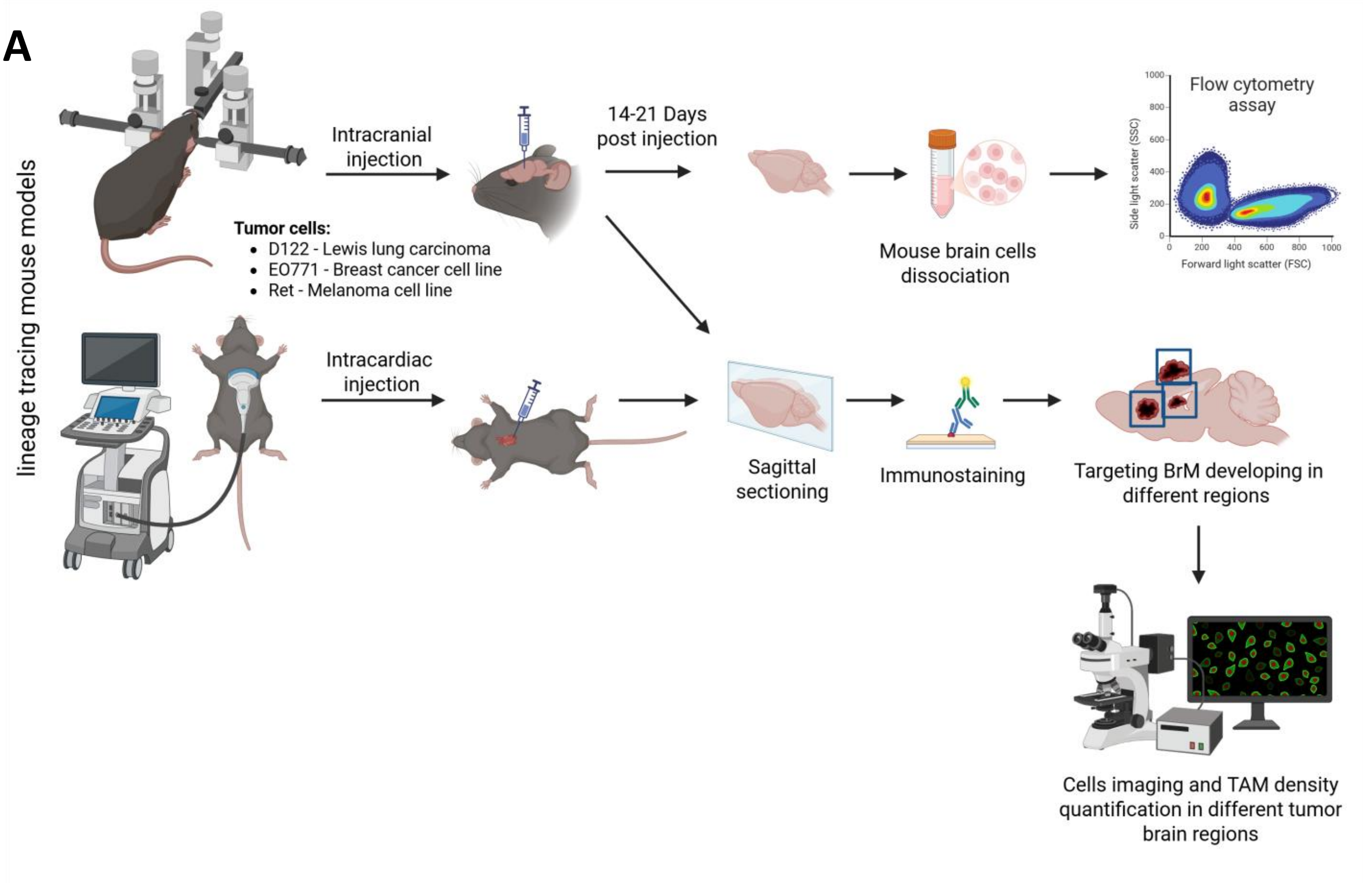

B

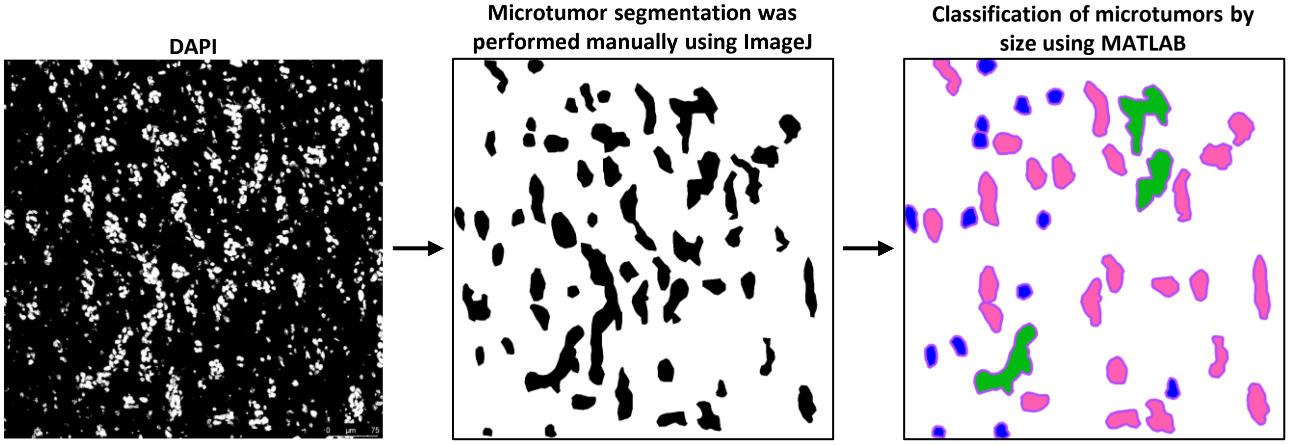

Small: 70-1,300 pixels (130-738.6 µm²)
Medium: 2,000-4,000 pixels (1,136-2,272 µm²)
Large: 6,000-10,000 pixels (3,409-6,818 µm²)

Fig. S2

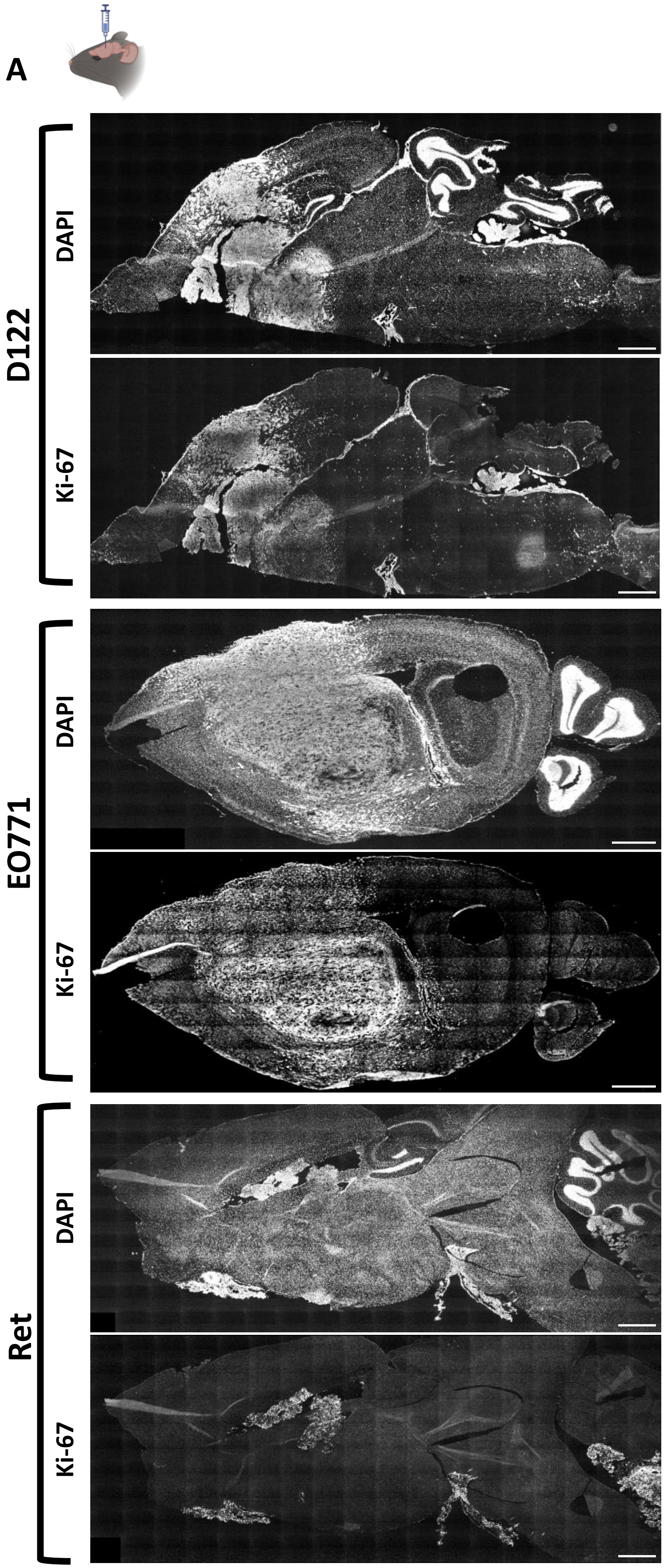

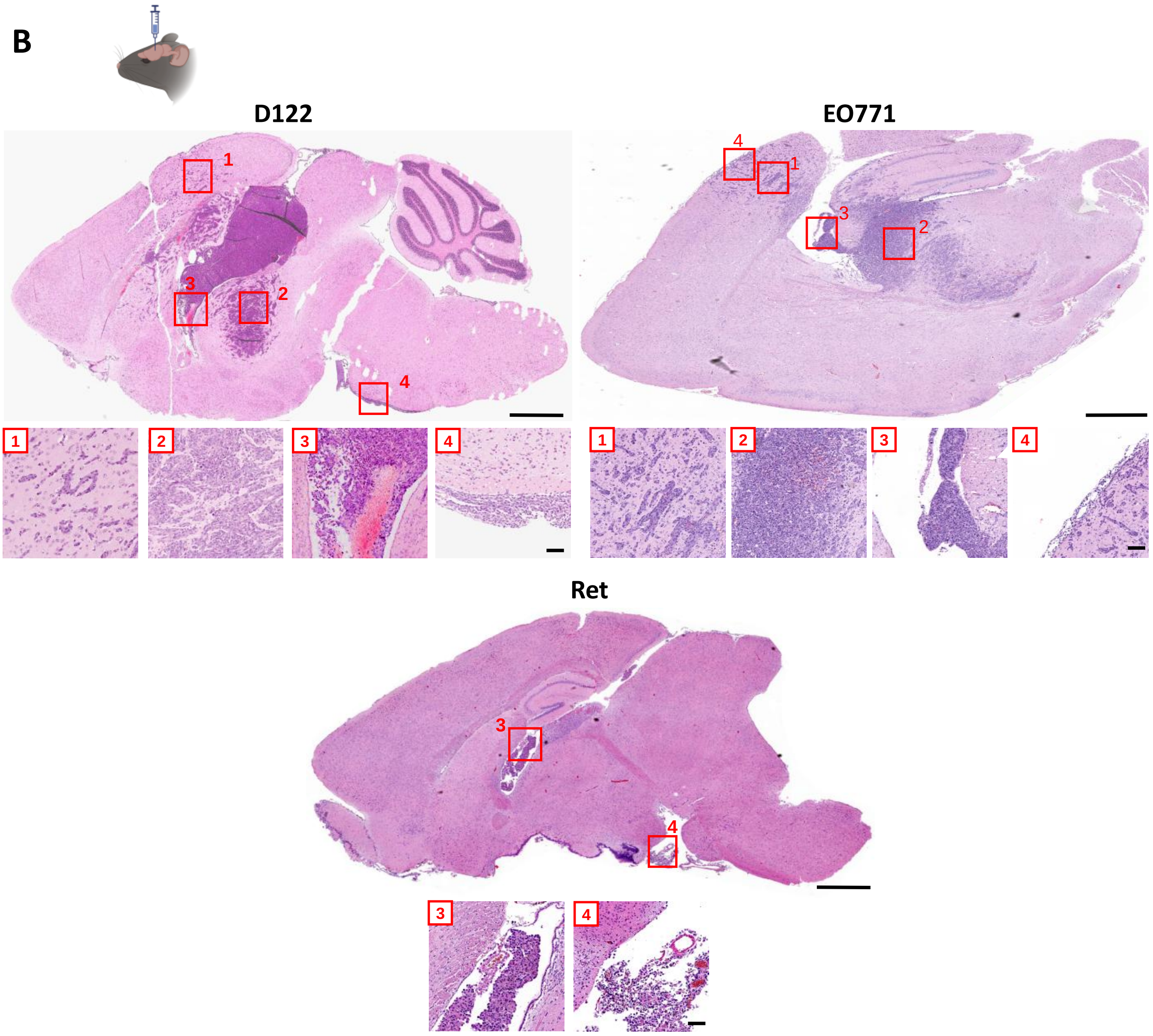
B
D122
EO771
Ret
1
2
3
4

Fig. S3

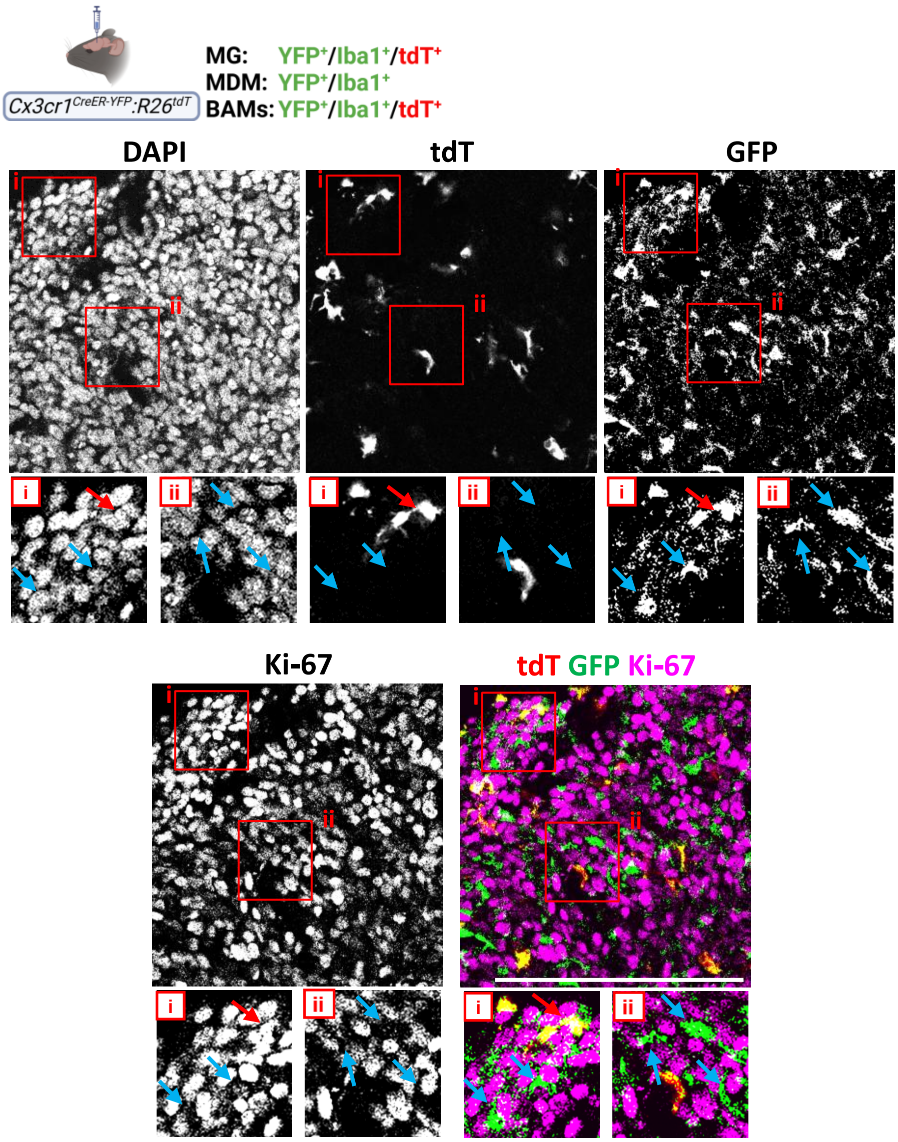
Cx3cr1CreER-YFP:R26tdT
MG: YFP+/Iba1+/tdT+
MDM: YFP+/Iba1+
BAMs: YFP+/Iba1+/tdT+
DAPI
tdT
GFP
Ki-67
tdT GFP Ki-67
i
ii

Fig. S4

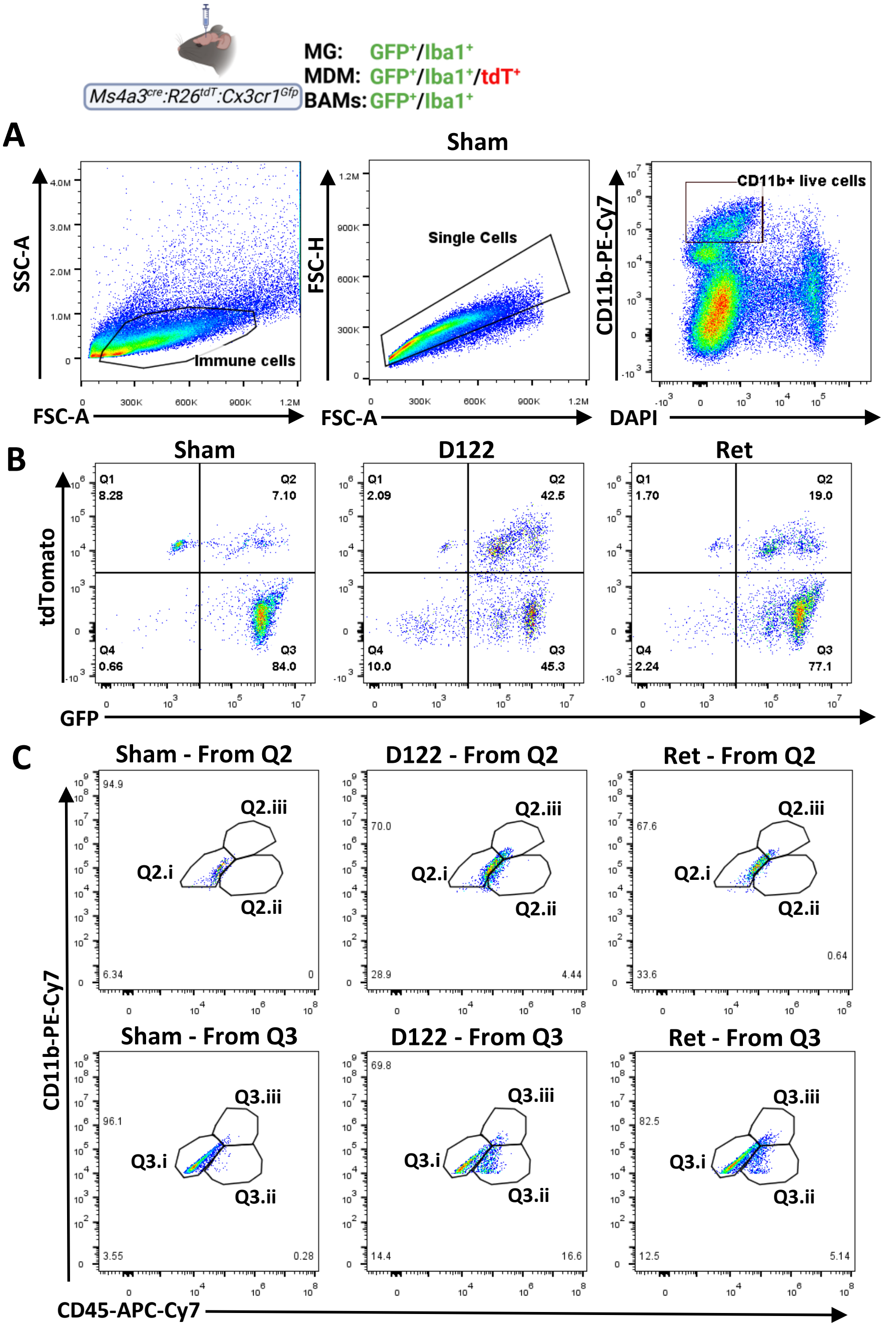

Ms4a3cre:R26tdT:Cx3cr1Gfp
MG: GFP+/Iba1+
MDM: GFP+/Iba1+/tdT+
BAMs: GFP+/Iba1+
A
Sham
SSC-A
FSC-A
Immune cells
FSC-H
Single Cells
CD11b-PE-Cy7
DAPI
CD11b+ live cells
B
Sham
D122
Ret
tdTomato
GFP
Q1 8.28
Q2 7.10
Q4 0.66
Q3 84.0
Q1 2.09
Q2 42.5
Q4 10.0
Q3 45.3
Q1 1.70
Q2 19.0
Q4 2.24
Q3 77.1
C
Sham - From Q2
D122 - From Q2
Ret - From Q2
Q2.i
Q2.ii
Q2.iii
Sham - From Q3
D122 - From Q3
Ret - From Q3
Q3.i
Q3.ii
Q3.iii
CD11b-PE-Cy7
CD45-APC-Cy7

Fig. S5

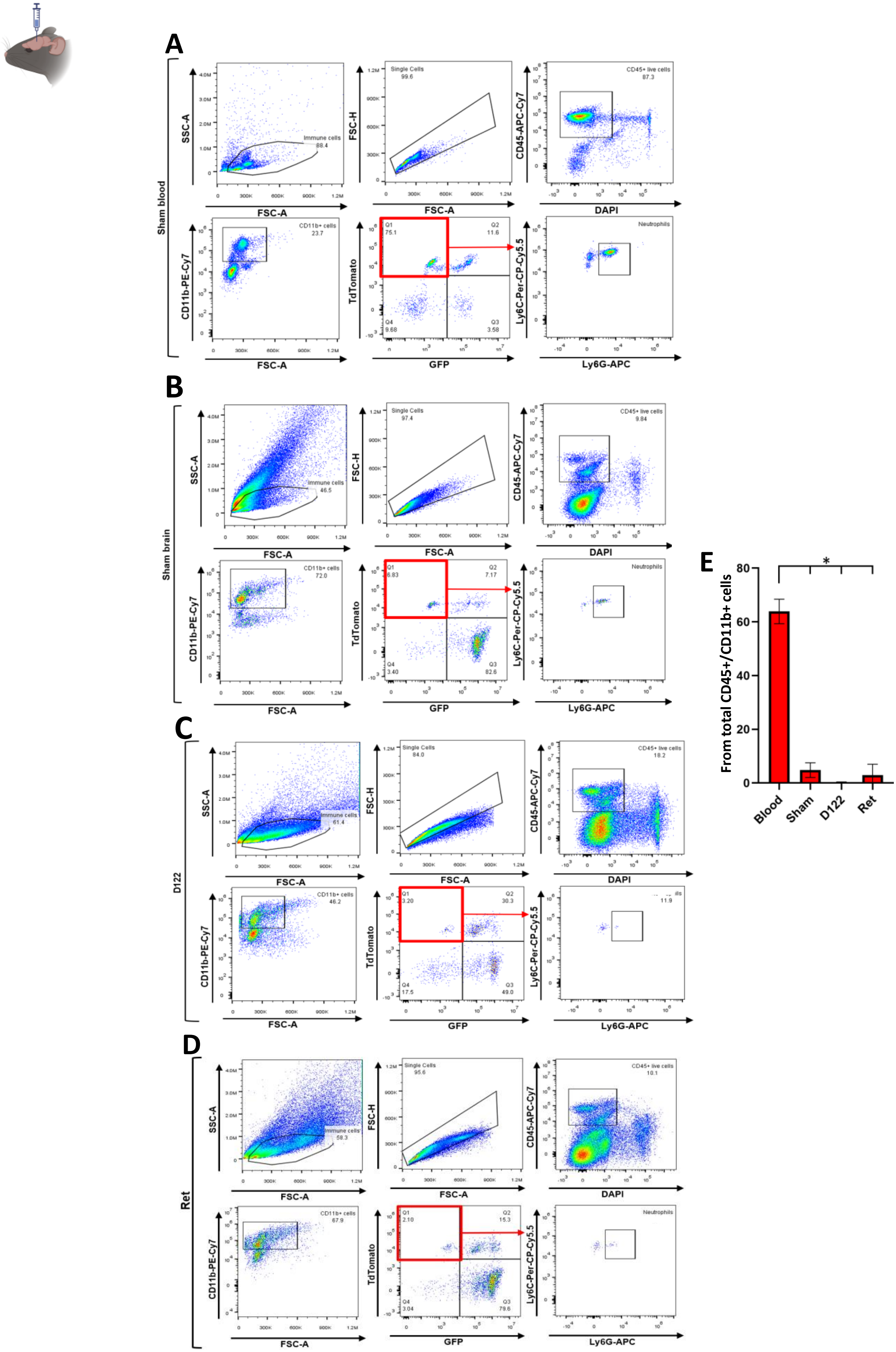

A
Sham blood
SSC-A
FSC-A
Immune cells
88.4
FSC-H
Single Cells
99.6
CD45-APC-Cy7
DAPI
CD45+ live cells
87.3
CD11b-PE-Cy7
CD11b+ cells
23.7
TdTomato
GFP
Q1
75.1
Q2
11.6
Q3
3.58
Q4
9.68
Ly6C-Per-CP-Cy5.5
Ly6G-APC
Neutrophils
B
Sham brain
Immune cells
46.5
Single Cells
97.4
CD45+ live cells
9.84
CD11b+ cells
72.0
Q1
6.83
Q2
7.17
Q3
82.6
Q4
3.40
Neutrophils
C
D122
Immune cells
61.4
Single Cells
84.0
CD45+ live cells
18.2
CD11b+ cells
46.2
Q1
3.20
Q2
30.3
Q3
49.0
Q4
17.5
11.9
D
Ret
Immune cells
58.3
Single Cells
95.6
CD45+ live cells
10.1
CD11b+ cells
67.9
Q1
2.10
Q2
15.3
Q3
79.6
Q4
3.04
Neutrophils
E
From total CD45+/CD11b+ cells
*
Blood
Sham
D122
Ret

Fig. S6

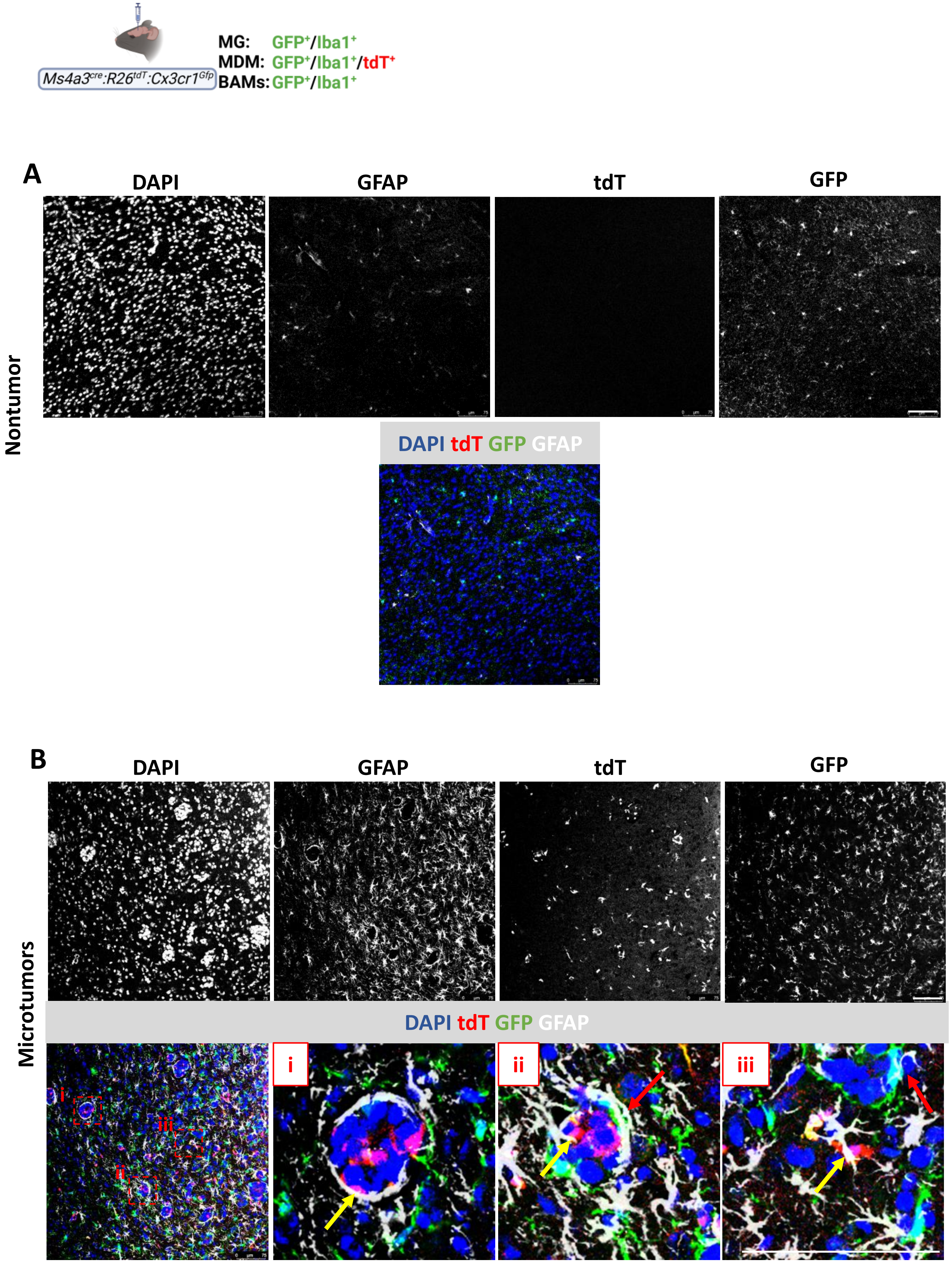
Ms4a3cre:R26tdT:Cx3cr1Gfp
MG: GFP+/Iba1+
MDM: GFP+/Iba1+/tdT+
BAMs: GFP+/Iba1+
A
DAPI
GFAP
tdT
GFP
Nontumor
DAPI tdT GFP GFAP
B
DAPI
GFAP
tdT
GFP
Microtumors
DAPI tdT GFP GFAP
i
ii
iii

Fig. S7

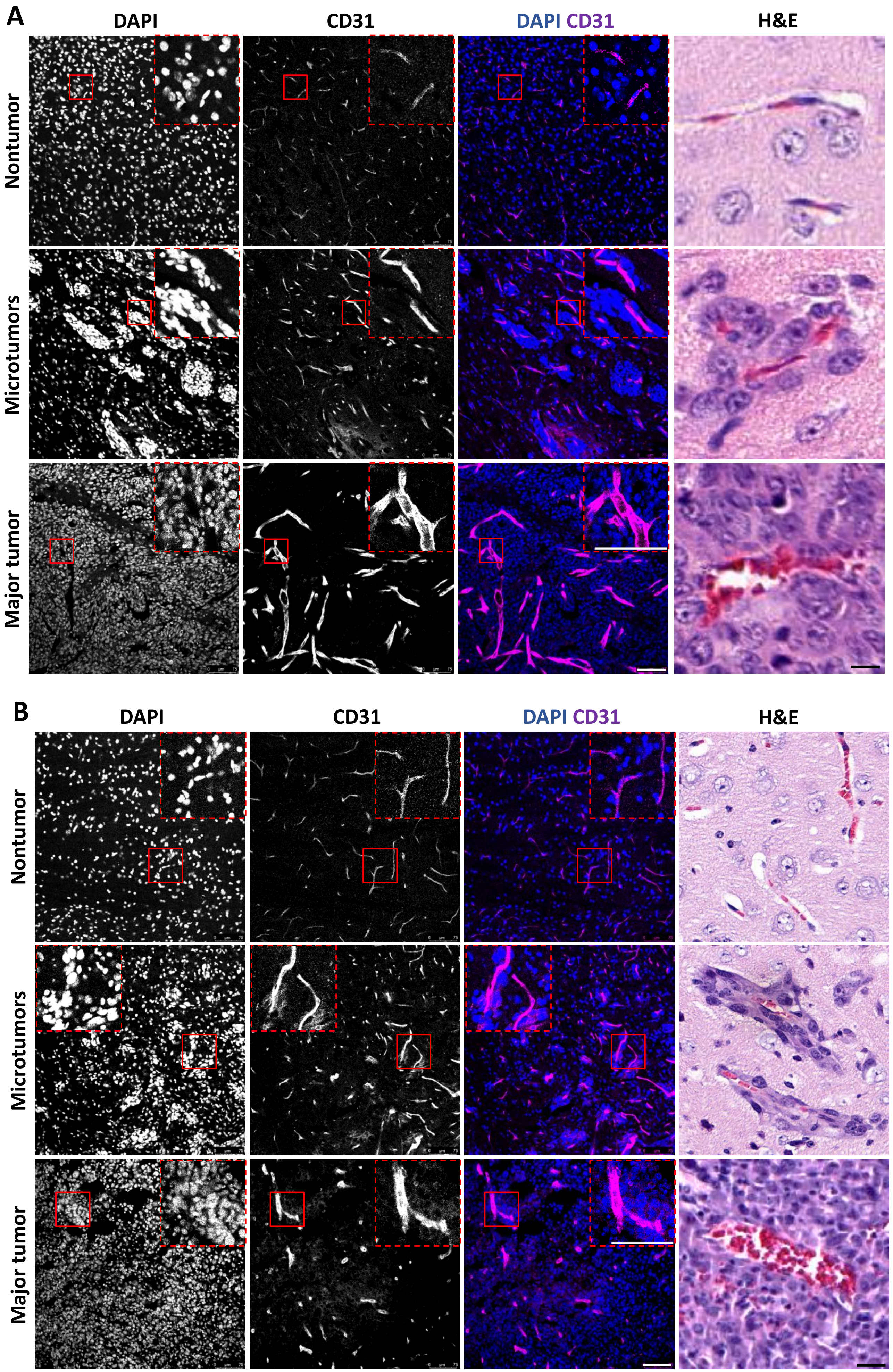
A
DAPI
CD31
DAPI CD31
H&E
Nontumor
Microtumors
Major tumor
B
DAPI
CD31
DAPI CD31
H&E
Nontumor
Microtumors
Major tumor

Fig. S8

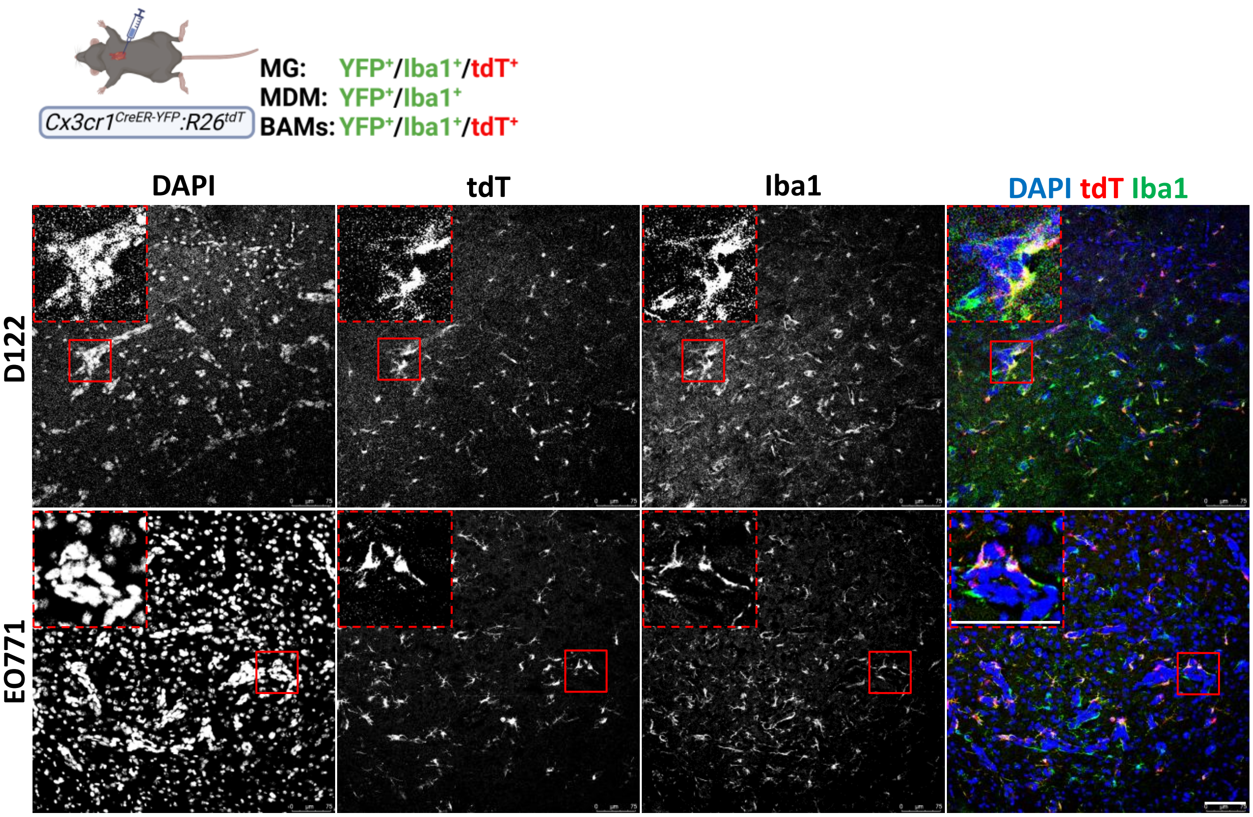

MG: YFP+/Iba1+/tdT+
MDM: YFP+/Iba1+
BAMs: YFP+/Iba1+/tdT+
Cx3cr1CreER-YFP:R26tdT
DAPI
tdT
Iba1
DAPI tdT Iba1
D122
EO771

Fig. S9

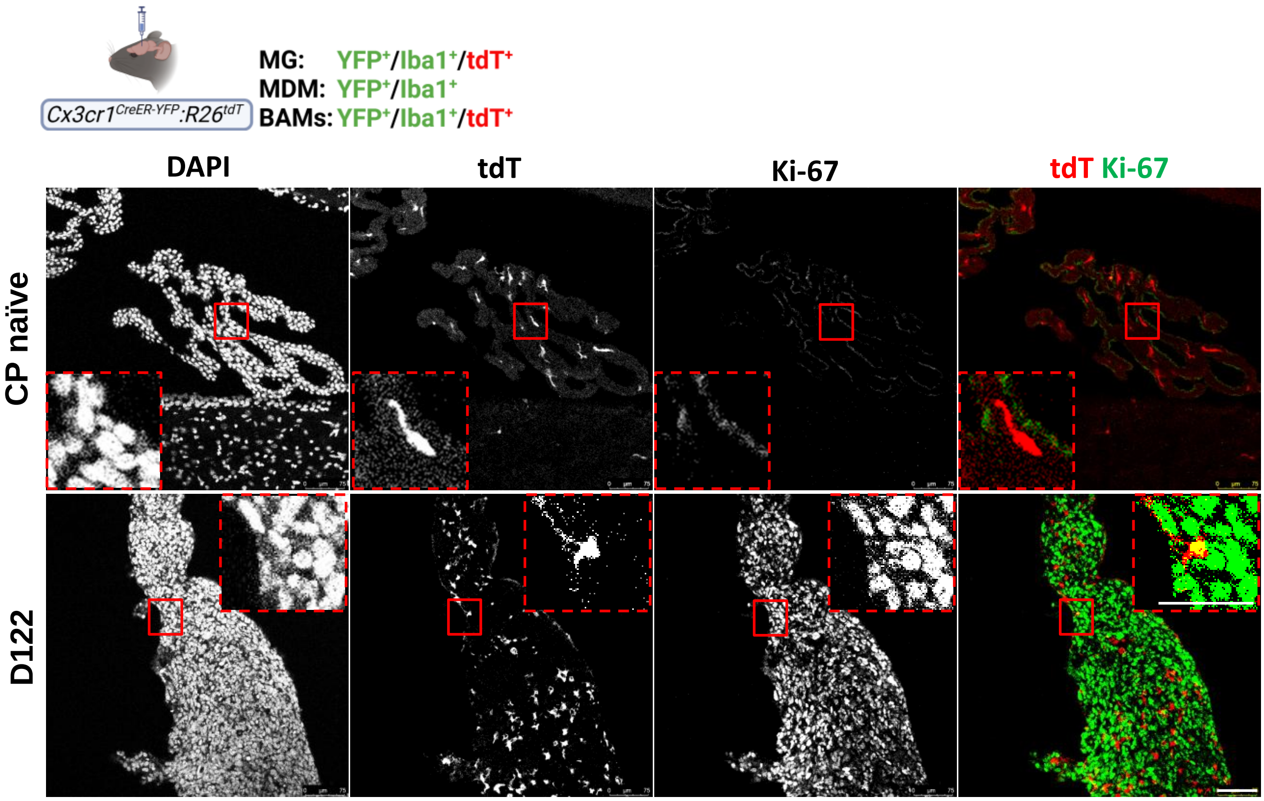

Cx3cr1CreER-YFP:R26tdT
MG: YFP+/Iba1+/tdT+
MDM: YFP+/Iba1+
BAMs: YFP+/Iba1+/tdT+
DAPI
tdT
Ki-67
tdT Ki-67
CP naïve
D122

Fig. S10

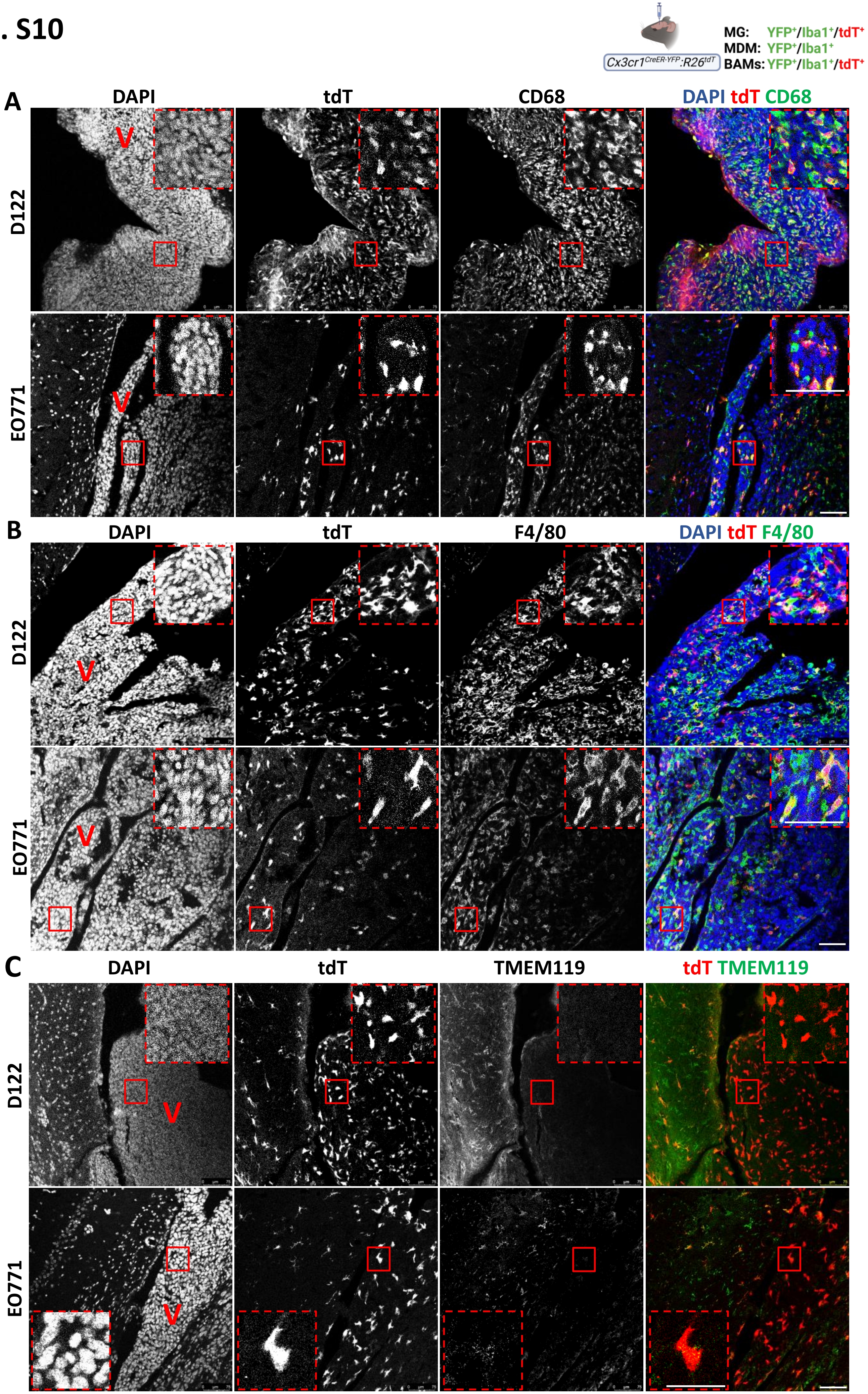

MG: YFP+/Iba1+/tdT+
MDM: YFP+/Iba1+
BAMs: YFP+/Iba1+/tdT+
Cx3cr1CreER-YFP:R26tdT
A
DAPI
tdT
CD68
DAPI tdT CD68
D122
EO771
V
B
DAPI
tdT
F4/80
DAPI tdT F4/80
D122
EO771
V
C
DAPI
tdT
TMEM119
tdT TMEM119
D122
EO771
V

Fig. S11

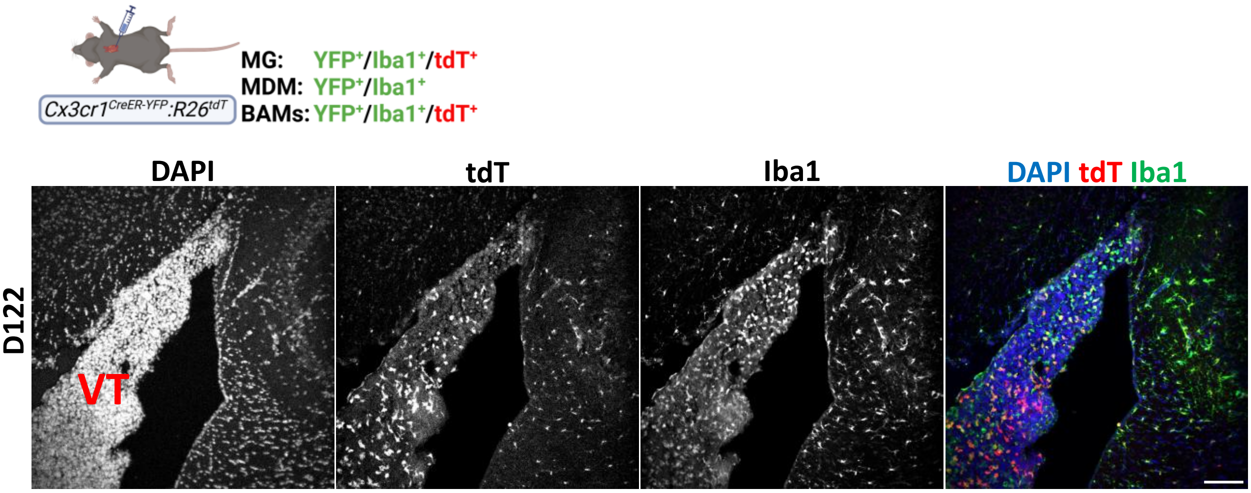

Cx3cr1CreER-YFP:R26tdT
MG: YFP+/Iba1+/tdT+
MDM: YFP+/Iba1+
BAMs: YFP+/Iba1+/tdT+
DAPI
tdT
Iba1
DAPI tdT Iba1
D122
VT

Fig. S12

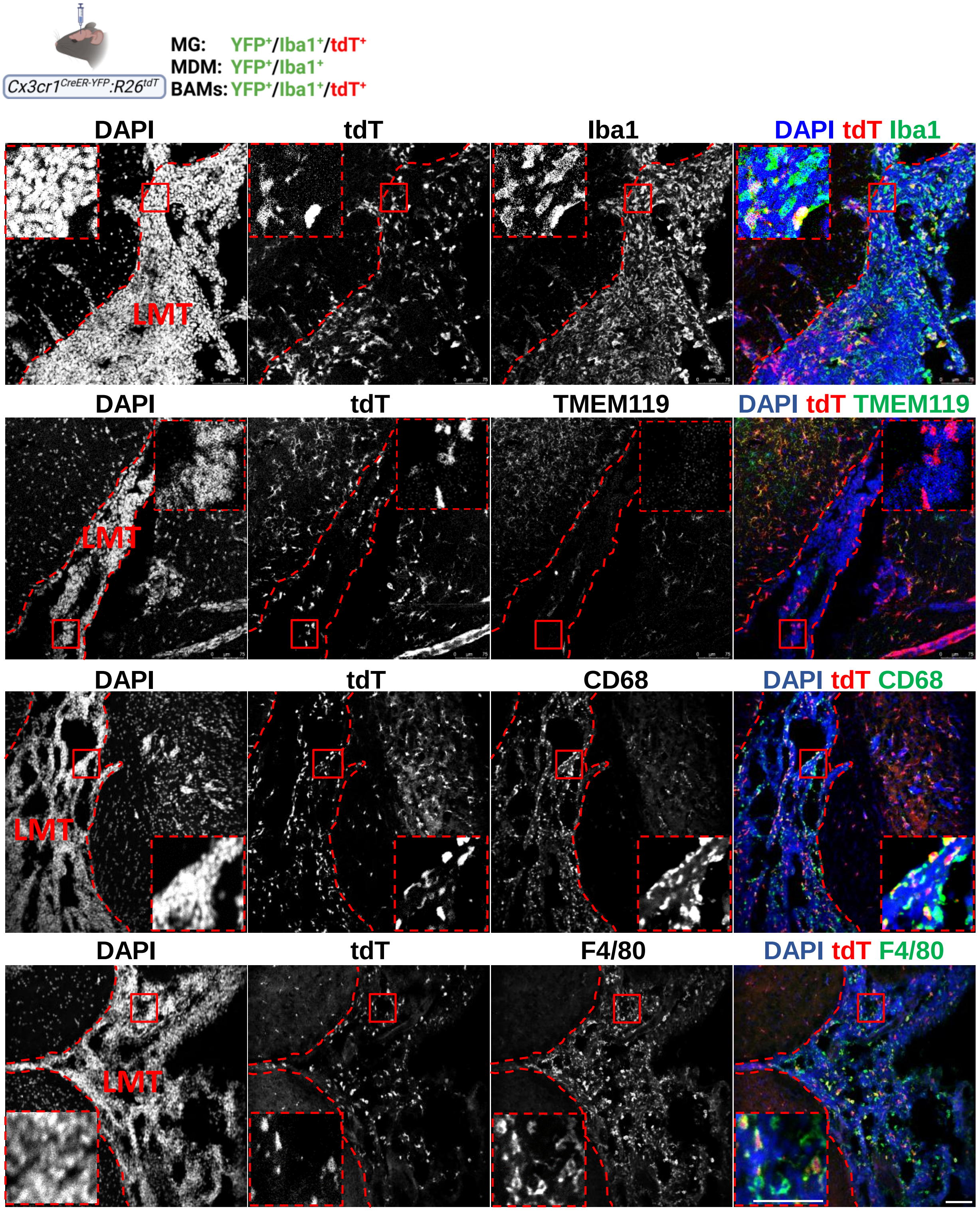

Cx3cr1CreER-YFP:R26tdT
MG: YFP+/Iba1+/tdT+
MDM: YFP+/Iba1+
BAMs: YFP+/Iba1+/tdT+
DAPI
tdT
Iba1
DAPI tdT Iba1
LMT
DAPI
tdT
TMEM119
DAPI tdT TMEM119
LMT
DAPI
tdT
CD68
DAPI tdT CD68
LMT
DAPI
tdT
F4/80
DAPI tdT F4/80
LMT

Fig. S13

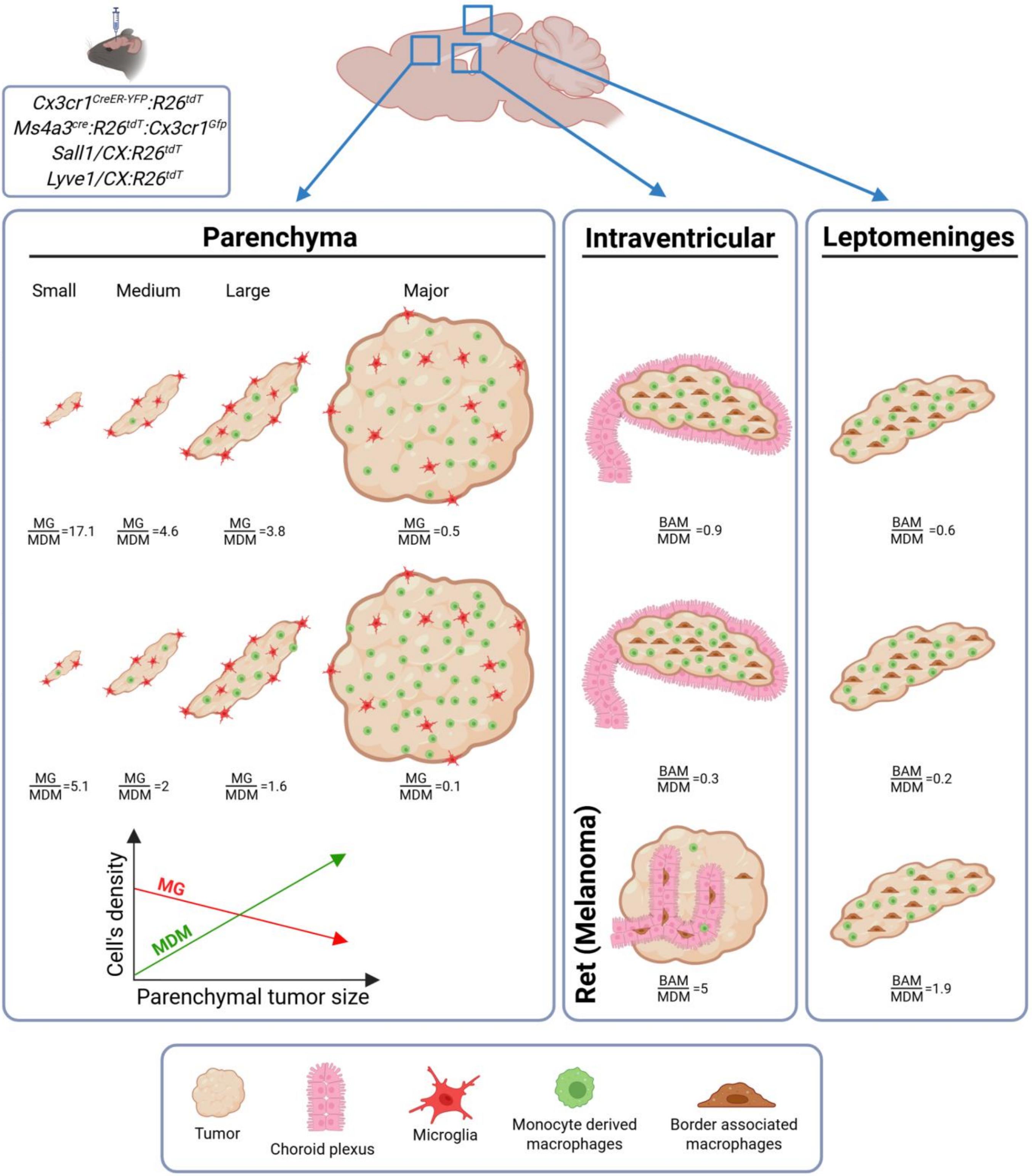

Cx3cr1CreER-YFP:R26tdT
Ms4a3cre:R26tdT:Cx3cr1Gfp
Sall1/CX:R26tdT
Lyve1/CX:R26tdT
Parenchyma
Intraventricular
Leptomeninges
Small
Medium
Large
Major
D122 (Lung)
MG/MDM =17.1
MG/MDM =4.6
MG/MDM =3.8
MG/MDM =0.5
BAM/MDM =0.9
BAM/MDM =0.6
E0771 (Breast)
MG/MDM =5.1
MG/MDM =2
MG/MDM =1.6
MG/MDM =0.1
BAM/MDM =0.3
BAM/MDM =0.2
Ret (Melanoma)
BAM/MDM =5
BAM/MDM =1.9
Cell's density
MG
MDM
Parenchymal tumor size
Tumor
Choroid plexus
Microglia
Monocyte derived macrophages
Border associated macrophages
