# Supplementary material for: Brain metastases exhibit distinct spatial patterns of resident and infiltrating macrophages
Source: Cell Death Discov. 2026 Apr 1;12:211. doi: 10.1038/s41420-026-03084-0 (PMC13168416; doi:10.1038/s41420-026-03084-0)
Supplement: Supplementary file 2 — Supplementary Figure Legends [file 41420_2026_3084_MOESM2_ESM.docx]

**Brain Metastases Exhibit Distinct Spatial Patterns of Resident and Infiltrating Macrophages**

Avinoam Ratzabi, Itai M. Caspit, Irina Telechi, Jung-Seok Kim, [Hananya Vaknine](https://pubmed.ncbi.nlm.nih.gov/?sort=date&size=100&term=Vaknine+H&cauthor_id=22700727), Pablo Blinder, Steffen Jung^c^ and Reuven Stein

**Supplementary Materials**

**Supplementary Figure legends**

**Supplementary Fig. S1.** **Experimental workflow and image analysis pipeline.**(A) Schematic overview of the experimental workflow. Tumor cells (D122 – Lewis lung carcinoma, E0771 – breast cancer, and Ret – melanoma) were injected either intracranially or intracardially into different lineage tracing mice as indicated in the text. 14-21 days post-injection, brains were collected for two parallel analyses: (i) Flow cytometry to assess the properties of different myeloid cell populations in sham- and tumor cells- injected mice. (ii) Immunostaining of sagittal sections to visualize and quantify brain metastases (BrM) size and tumor-associated macrophages (TAM) distribution and content in distinct brain compartments using fluorescence microscopy. (B) Image analysis workflow. Parenchymal microtumors - microscopic images were processed as follows: nuclei (DAPI) were visualized, and microtumors were manually segmented using ImageJ. The segmented regions were then analyzed and classified by size (small, medium, large) using a custom MATLAB script. The size thresholds and classification criteria used for microtumors categorization are detailed in the Materials and Methods section. To assess cell density in parenchymal major tumors, ventricular tumors, and leptomeningeal tumors, the entire tumor area and its associated TAM were manually determined and counted using ImageJ.

**Supplementary Fig. S2.** **DAPI staining is a reliable method for BrM detection**

(A) Colocalization of DAPI and Ki-67 IF signals in BrM. D122, EO771, or Ret cells were intracranially injected into *Cx3cr1^CreER-YFP^:R26^tdT^* mice. Representative scanned sagittal sections show DAPI (upper panels) and Ki-67 (lower panels) from the same fields. Scale bar: 1000 µm. **(B)** H&E assessment of lung cancer, breast cancer, and melanoma BrM colonization. D122, EO771, or Ret cells were intracranially injected into *Cx3cr1^CreER-YFP^:R26^tdT^* mice. Representative H&E-stained sagittal sections illustrate four BrM growth patterns: parenchymal [microtumors (1) and major tumors (2)], ventricular (3), and leptomeningeal (4). Red boxes mark regions enlarged below. Scale bars, 1000 µm (overview) and 100 µm (enlargements).

**Supplementary Fig. S3. Minimal proliferation of monocyte-derived macrophages within brain metastases.** *Cx3cr1^CreER-YFP^:R26^tdT^* mice were intracranially injected with D122 tumor cells. Sagittal brain sections were stained with DAPI, anti-GFP, and anti-Ki-67 antibodies. Representative confocal IF images show DAPI, tdT, GFP, and Ki-67 signals in the same field, along with a merged image of tdT, GFP, and Ki-67 channels. Enlarged views of the boxed regions (i and ii) are shown below. Scale bar, 75 μm. Red arrows indicate proliferating MG (tdT⁺/GFP⁺/Ki-67⁺), whereas blue arrows indicate non-proliferating MDM (tdT⁻/GFP⁺/Ki-67⁻). Quantification revealed that only 1.13% of total MDM (~4,000 cells, n = 4) were Ki-67⁺, demonstrating that MDM proliferation within brain metastases is negligible.

**Supplementary Fig. S4.** **Gating strategy for the flow cytometric characterization of macrophage subpopulations in BrM-bearing brains**. Brains from *Ms4a3^cre^:R26^tdT^:Cx3cr1^Gfp^* mice injected with D122 or Ret cells, or subjected to sham treatment, were processed for flow cytometry at 21, 14, and 21 dpi, respectively. (A-C) Representative dot plots of the gating strategy. (A) Immune cells were first gated on SSC-A vs FSC-A, then singlets (FSC-H vs FSC-A), and finally CD11b^+^ live cells (example shown for sham). (B) CD11b^+^ cells were then gated on tdT and GFP. (C) MDM (tdT^+^/GFP^+^, Q2 in B) and MG (tdT^–^/GFP^+^, Q3 in B) were each subsequently subdivided by CD11b and CD45 expression into three subpopulations: (i) CD45^int^/CD11b^int^, (ii) CD45^hi^/CD11b^int^, and (iii) CD45^hi^/CD11b^hi^.

**Supplementary Fig. S5. Flow cytometric analysis of neutrophils.** Brains from sham-, D122-, or Ret-injected *Ms4a3^cre^:R26^tdT^:Cx3cr1^Gfp^* mice, as well as blood from sham-injected mice, were processed for flow cytometry. (A–D) Representative dot plots of the gating strategy for blood (A), sham brain (B), and D122- (C) or Ret- (D) BrM-containing brains. Immune cells were first gated on SSC-A vs FSC-A, followed by singlets (FSC-H vs FSC-A), CD45⁺ live cells, and then CD11b⁺ cells. Cells were further gated on tdT and GFP, after which neutrophils were identified based on Ly6G and Ly6C expression (Ly6G⁺/Ly6C⁺ cells). (E) Quantification of the percentage of neutrophils within the total myeloid population (CD45⁺/CD11b⁺). Data are presented as mean ± SEM (n = 4, 4, 5, and 3 for blood, sham, D122-, and Ret-injected mice, respectively).

**Supplementary Fig. S6.** **Spatial distribution of astrocytes in D122 BrM-bearing *Ms4a3^cre^:R26^tdT^:Cx3cr1^Gfp^* mice**. Sagittal sections were stained with DAPI together with anti-GFAP and anti-GFP antibodies. Representative confocal images of nontumor (A) and microtumors- containing regions (B) (n = 5–6 mice) show tdT, GFAP, and GFP signals individually (upper panels) and as merged images (lower panels) (DAPI: blue, tdT: red, GFP: green, GFAP: white). Enlarged regions in the merged image of microtumors show: (i) astrocytes (GFAP^+^) wrapping the microtumors and interacting with MDM (tdT^+^) in the microtumor surface and with the adjacent TA-MG (tdT^−^GFP^+^), (ii) astrocytes contacting microtumors at the tumor margin, and (iii) astrocytes interacting with MDM between microtumors. Yellow and red arrows indicate astrocytes interacting with MDM and MG, respectively. Scale bar: 75 µm.

**Supplementary Fig. S7.** **Blood vessels in D122 and EO771 parenchymal BrM**. Sagittal sections were stained with DAPI and anti-CD31 or with H&E. Confocal images (n = 6 mice) show DAPI and CD31 signals individually and merged (DAPI: blue, CD31: pink; left panels). Far-right panels show H&E. Insets in the IF images display enlarged vessel-containing regions. Note the degenerative morphology of BrM-associated vessels, particularly in major tumors. Scale bar: 75 µm.

**Supplementary Fig. S8.** **Spatial distribution of tumor-associated microglia and monocyte-derived macrophages in BrM established by intracardiac injection**. *Cx3cr1^CreER-YFP^:R26^tdT^* mice were intracardially injected with D122 or EO771 cells. Sagittal sections were stained and analyzed as in **Fig. 2A** (n = 4 mice). Insets are enlargements of regions outlined by red boxes. Scale bar: 75 µm.

**Supplementary Fig. S9.** **Proliferating tdT^+^ cells in ventricular BrMs in *Cx3cr1^CreER-YFP^:R26^tdT^* mice**. Sagittal sections of naïve and D122 BrM-containing brains were stained and analyzed as in **Fig. 5A**. Representative confocal images showing DAPI, tdT, and Ki-67 signals, individually and merged, depict a choroid plexus (CP) from a naïve mouse and a ventricular D122 BrM. Insets highlight a representative BrM region containing a proliferating tdT⁺ cell in the D122 BrM sample. Note that most Ki-67⁺ cells within the BrM region are metastatic cells (tdT^−^). **Scale bar:** 75 µm. Related to **Fig. 9D**. (n = 6 mice)

**Supplementary Fig. S10.** **Expression of marker proteins in tdT^+^ cells and monocytes derived macrophages within D122 and EO771 ventricular tumors in *Cx3cr1^CreER-YFP^:R26^tdT^* mice.** Sagittal sections from BrM containing brains were stained with DAPI and either anti-CD68 (A), anti-F4/80 (B), or anti-TMEM119 (C) antibodies. Representative confocal images of ventricular BrM tumor (VT)-containing regions are shown as in **Fig. 6A-C** (n = 6–8 mice). Scale bar: 75 µm.

**Supplementary Fig. S11.** **Distribution of *Cx3cr1*-expressing cells and monocytes-derived macrophages in ventricular D122 BrM generated by intracardiac injection**. Sagittal sections obtained from intracardiac injected *Cx3cr1^CreE-YFP^:R26^tdT^* mice were stained with DAPI and anti-Iba1. Representative confocal images of ventricular BrM tumor (VT)-containing regions are shown as in **Fig. 9B**. Note the presence of *Cx3cr1*-expressing cells tdT^+^/Iba1^+^ cells and MDM (tdT^−^/Iba1^+^ cells) within the tumor mass. Scale bar: 75 µm.

**Supplementary Fig. S12 Expression of marker proteins in tdT⁺ cells and monocytes-derived macrophages within D122 leptomeningeal BrM.** Sagittal sections containing leptomeningeal BrM from intracranially injected *Cx3cr1^CreER-YFP^:R26^tdT^* mice were stained with DAPI and anti-Iba1 together with TMEM119, anti-CD68, or anti-F4/80 antibodies. Representative confocal IF images (n = 5–6 mice) show tdT and the indicated marker protein signals as individual channels and merged images. Insets show enlarged views of boxed regions. Scale bar: 75 μm.

**Supplementary Fig. S13.** **Schematic summary illustrating compartment-specific alterations in tumor-associated macrophage composition across brain metastases**. Using complementary lineage-tracing mouse models and immunofluorescence, microglia (MG), monocyte-derived macrophages (MDM), and border-associated macrophages (BAM) were identified. Parenchymal tumors contained MG and MDM, while ventricular and leptomeningeal metastases comprised BAM and MDM. MG initially encircle parenchymal microtumors, followed by MDM infiltration and a shift toward MDM predominance during tumor growth. Distinct cancer origins showed different compartmental tropism and TAM composition, with lung and breast metastases (D122, EO771) forming within the choroid plexus and enriched in MDM/BAM, whereas Ret melanoma metastases developed outside it and contained only minimal MDM/BAM.
